# Cardiac structure discontinuities revealed by ex-vivo microstructural characterization. A focus on the basal inferoseptal left ventricle region

**DOI:** 10.1186/s12968-023-00989-y

**Published:** 2023-12-14

**Authors:** Pierre Cabanis, Julie Magat, Jairo Rodriguez-Padilla, Girish Ramlugun, Maxime Yon, Yann Bihan-Poudec, Nestor Pallares-Lupon, Fanny Vaillant, Philippe Pasdois, Pierre Jais, Pierre Dos-Santos, Marion Constantin, David Benoist, Line Pourtau, Virginie Dubes, Julien Rogier, Louis Labrousse, Michel Haissaguerre, Olivier Bernus, Bruno Quesson, Richard Walton, Josselin Duchateau, Edward Vigmond, Valéry Ozenne

**Affiliations:** 1 Univ. Bordeaux, CNRS, CRMSB, UMR 5536, Bordeaux, France; 2 Liryc, Electrophysiology and Heart Modeling Institute, Fondation Bordeaux Université, Pessac-Bordeaux, France; 3 Inria Epione Team, Université Côte d’Azur, Biot, France; 4 Centre de Neuroscience Cognitive, CNRS, Université Claude Bernard Lyon I, Villeurbanne, France; 5 Cardiology Department, Bordeaux University Hospital (CHU), Pessac, France; 6 Centre de Résonance Magnétique des Systèmes Biologiques, 2 Rue Dr Hoffmann Martinot, 33000 Bordeaux, France; 7 Present Address: CNRS, IMB, UMR5251, Talence, France

**Keywords:** Diffusion MRI, Diffusion tensor imaging, Tractography, Ex vivo heart, 3D cardiomyocytes architecture, Right ventricular insertion point, Ventricular junction

## Abstract

**Background:**

While the microstructure of the left ventricle (LV) has been largely described, only a few studies investigated the right ventricular insertion point (RVIP). It was accepted that the aggregate cardiomyocytes organization was much more complex due to the intersection of the ventricular cavities but a precise structural characterization in the human heart was lacking even if clinical phenotypes related to right ventricular wall stress or arrhythmia were observed in this region.

**Methods:**

MRI-derived anatomical imaging (150 µm^3^) and diffusion tensor imaging (600 µm^3^) were performed in large mammalian whole hearts (human: N = 5, sheep: N = 5). Fractional anisotropy, aggregate cardiomyocytes orientations and tractography were compared within both species. Aggregate cardiomyocytes orientation on one ex-vivo sheep whole heart was then computed using structure tensor imaging (STI) from 21 µm isotropic acquisition acquired with micro computed tomography (MicroCT) imaging. Macroscopic and histological examination were performed. Lastly, experimental cardiomyocytes orientation distribution was then compared to the usual rule-based model using electrophysiological (EP) modeling. Electrical activity was modeled with the monodomain formulation.

**Results:**

The RVIP at the level of the inferior ventricular septum presented a unique arrangement of aggregate cardiomyocytes. An abrupt, mid-myocardial change in cardiomyocytes orientation was observed, delimiting a triangle-shaped region, present in both sheep and human hearts. FA’s histogram distribution (mean ± std: 0.29 ± 0.06) of the identified region as well as the main dimension (22.2 mm ± 5.6 mm) was found homogeneous across samples and species. Averaged volume is 0.34 cm^3^ ± 0.15 cm^3^. Both local activation time (LAT) and morphology of pseudo-ECGs were strongly impacted with delayed LAT and change in peak-to-peak amplitude in the simulated wedge model.

**Conclusion:**

The study was the first to describe the 3D cardiomyocytes architecture of the basal inferoseptal left ventricle region in human hearts and identify the presence of a well-organized aggregate cardiomyocytes arrangement and cardiac structural discontinuities. The results might offer a better appreciation of clinical phenotypes like RVIP-late gadolinium enhancement or uncommon idiopathic ventricular arrhythmias (VA) originating from this region.

**Supplementary Information:**

The online version contains supplementary material available at 10.1186/s12968-023-00989-y.

## Introduction

The growing number of imaging technologies that have emerged over the last decades has greatly extended our knowledge of cardiac microstructure, and given detailed information about 3D cardiomyocyte architecture [1]. Nevertheless, there is still a gap in our understanding of both the course of events of pathophysiological processes like cardiac remodeling [2], and the link with clinical phenotypes [3]. Recently, some concerns have been raised regarding the interpretation of the presence of late gadolinium enhancement (LGE) at the right ventricle attachment or insertion point (RVIP) [4–9].

The origin of the LGE pattern has not been extensively described in the literature. The existing studies [10–12] examined it using histopathology of human postmortem hearts in the context of hypertrophic cardiomyopathy (HCM) or pulmonary arterial hypertension (PAH). More recently, Friedberg et al. [13] investigated the role of regional myocardial remodeling in rat or rabbit in the context of PAH using echocardiography and histopathology. All these studies reported the presence of myocardial disarray, expanded extracellular space, disorderly arranged myocytes, or an increase of regional fibrosis. The only published 3D descriptions were two studies that quantified either myoarchitectural disarray in HCM rats using high-resolution episcopic microscopy [14] or infarcted myocardium using tractrography processing in patients [15]. In contrast to the above-mentioned studies, we recently reported an abrupt mid-myocardial change in cardiomyocytes orientation at the RVIP in ex-vivo large mammalian samples [16] and did not report the presence of myoarchitectural disarray but the study was limited to a few sheep samples.

The purpose of the present work is to characterize in ex-vivo large mammalian (human and sheep) hearts, the basal inferoseptal (BIS) cardiomyocyte orientation and assess its functional impact on electrophysiology. In particular, it aims to detail the 3D structure organization by identifying the cardiomyocyte organization with high-resolution diffusion MRI at 600 µm^3^, and X-ray imaging at 21 µm^3^ and 2D histology. Experimental characterization of the microstructure organization is also largely used in realistic or patient-specific models for biomechanical, or electrophysiological models. Recently, Doste et al. [17] underlined the discrepancy between cardiomyocyte orientation distributions computed by rule-based models with histological or MRI data in the IVS due to the dual-layer organization [18], and in the RVIP. Thus, to expand the scope of the result, based on the observed human experimental data, we model the effects of cardiomyocyte orientation on cardiac electrophysiology (EP), computing local activation times and pseudo-ECGs. Lastly, we discuss the hypothetical structural determinism relating aggregate cardiomyocyte orientation with the presence of focal LGE in this area.

## Methods

### Sample preparation

All hearts were fixed with a solution containing formalin (10%) and a gadolinium-based contrast agent (Dotarem, Guerbet, Paris, France) at 0.2% of total volume of perfusion, by retrograde perfusion from the aorta. Sample preparation was performed as previously described [16, 18–20].

### Human samples

Human samples (N = 5) were derived from the human donor program (providing access to heart samples from patients under cerebral death for scientific research purposes) approved by the ‘‘Agence Française de la Biomedecine’’ and with a written informed consent of the patient’s family. The experiment was conducted in accordance with the declaration of Helsinki and the institutional ethics committee. Five hearts (1 male and 4 females, 71 ± 15.5 years old) were used in the study. A brief summary of patient characteristics is available in Table 1. Hearts were reported with cardiac pathology and not eligible for cardiac transplantation. Donors of hearts #1 and #2 had a history of prior myocardial infarction, donor #3 had a history of mitral valve prolapse and donor #4 had a history of hypertensive cardiomyopathy and atrial fibrillation. For one heart (#5), no significant cardiac pathology was revealed during the clinical evaluation performed before explanting the heart [20]. This heart was thus labeled “control”.

**Table 1:** Summary of the key characteristic of the ex-vivo hearts

Heart n°	Age [y]	Sex	Dimensions [cm × cm × cm]	Cause of death
#S1	2	F	7.8 × 5.3 × 10.5	
#S2	2	F	7.6 × 5.3 × 10.0	
#S3	2	F	7.5 × 5.1 × 9.9	
#S4	2	F	8.4 × 6.3 × 9.8	
#S5	2	F	8.2 × 6.3 × 10.6	
#S6	10	F	6.3 × 5.2 × 6.7	
#H1	53	F	10.9 × 8.0 × 14.1	Hemorrhagic stroke
#H2	56	M	8.6 × 9.4 × 10.7	Cardiovascular accident, cerebral anoxia
#H3	82	F	8.2 × 10.1 × 11.1	Hemorrhagic stroke
#H4	83	F	10.1 × 8.1 × 11.4	Head trauma
#H5	83	F	8.4 × 7.4 × 12.1	Hemorrhagic stroke

The cause of death of the donor is also indicated. S: Sheep; H: Human; F: Female; M: Male

### Sheep samples for MRI and MicroCT

Sheep samples (N = 6; 5 for MRI, 1 for MicroCT) and protocols used in this study were approved by the Animal Research Ethics Committee (CEEA—050 Comité d’éthique pour l’expérimentation animale Bordeaux) in accordance with the European rules for animal experimentation. Hearts were explanted via sternal thoracotomy under general anesthesia using the following protocol. In brief, anesthesia was induced with an intravenous bolus of ketamine (15 mg/kg) and midazolam (1.5 mg/kg). Animals were then intubated and ventilated and received an injection of heparin (2.5 mg/kg). Anesthesia was maintained with ketamine and midazolam (40 mg kg^−1^ h^−1^ and 2 mg kg^−1^ h^−1^, respectively). The thorax was opened, and blood from each animal was collected via the introduction of an 8-Fr sheath into the right jugular vein. Heparin (15 mg/l) was added in the reservoir to avoid coagulation. Sheep were euthanized by intraperitoneal injection with sodium pentobarbital (35 mg/kg) and the hearts were quickly excised. The aorta was cannulated and perfused with cold cardioplegic (4 °C) solution supplemented with heparin (5 U/ml). The hearts were mostly arrested in the systolic phase (#S1-S4) with the exception of sample #S5 which was arrested in a cardiac phase closer to diastole. The myocardium is also relaxed in comparison to in-vivo state due to this first cardioplegic flushing before formalin fixation macroscopic examination was performed to verify the absence of overt cardiac disease.

### Additional protocol for microCT

A novel tissue preparation technique to alleviate background photon attenuation was performed using a tissue air-drying approach [21]. In this case, left and right coronary ostia were cannulated individually for optimal tissue perfusion. The #S6 heart (N = 1) was perfused with phosphate-buffered saline (PBS, pH = 7.4) followed by ethylenediaminetetraacetic acid (EDTA) [10 mM] solution with Papaverine [30 μM]. The heart was fixed 2 h with 4% formaldehyde, rinsed three times for 20 min in PBS and then dehydrated using ethanol and perfused with hexamethyldisilazane (HMDS) to prevent tissue deformation. Finally, the heart was hung to air-dry under the fume hood and inside small containers to reduce airflow for 7 days.

### Data acquisition

#### MRI acquisition

All experiments were performed at 9.4T with an inner bore size of 30 cm (BioSpin MRI; Bruker, Ettlingen, Germany) equipped with 300 mT/m gradient insert of 200 mm inner diameter adapted for large samples. Images were acquired using a dedicated radiofrequency volume array coil with seven elements in transmit and receive. All MRI scans and experiment acquisitions were similar to those described previously in [16, 18–20].

#### Diffusion-weighted (DW) imaging

A 3D DW spin-echo sequence was used to acquire DW images (TE/TR: 22/500 ms, acquisition time: 24 h). The DW dataset consisted of six noncollinear diffusion encoding directions acquired with a b value of 1000 s/mm^2^ with an isotropic spatial resolution of 600 µm^3^. The same parameters were set for each acquisition except for the field of view which was adjusted for each sample.

A 2D DW spin-echo sequence was used to acquire DW images with six noncollinear diffusion encoding directions to test the influence of b-value. The slice was positioned in SA view in mid-ventricular region on sheep sample #S2. 5 acquisitions (TE/TR = 22/500 ms, 5 averages, acquisition time: 24′13″) were done at 0.6 × 0.6 × 1 mm^3^ voxel resolution with b-values ranging at 100, 350, 500, 750 and 1000 s/mm^2^.

#### Anatomical imaging

3D anatomical image was acquired with a gradient echo sequence (TE/TR: 9/30 ms, acquisition time: 18 h) at an isotropic resolution of 150 µm^3^. The resulting volume is referred to as the anatomical image.

#### MicroCT acquisitions

Heart #S6 was imaged using microCT (SkyScan 1276, Bruker, Belgium). X-ray transmission images were acquired using X-ray source energies of 55 kV and 150 μA, and an aluminum plate of thickness 0.5 mm. An average of three acquisitions were captured at rotation steps incrementing by 0.18° over 360°. The total acquisition time was 8 h. Short-axis (SA) images were obtained by 3D tomographic reconstruction of the raw images using the cone-beam FDK11 algorithm using the NRecon software (Bruker). Images were reconstructed with an isotropic voxel resolution of 21 μm. The matrix size was 1801 × 1344 × 3872 for a corresponding field of view of 38 × 28 × 81 mm^3^.

### Image analysis

#### MRI diffusion tensor estimation

Each DW image was up-sampled by a factor of 2 using trilinear interpolation to reach a voxel size of 0.3 × 0.3 × 0.3 mm^3^. Diffusion tensor calculations were performed prior to any registration to avoid difficult transformations of the diffusion encoding matrix. Diffusion tensor maps [eigenvalues: λ1, λ2, λ3, apparent diffusion coefficient, fractional anisotropy (FA), and color-coded FA (cFA), also known as red–green–blue colormap] were obtained with MRtrix3 software (https://www.mrtrix.org) [22]. The first diffusion tensor eigenvector v1 was associated with the main aggregate cardiomyocytes orientation. To obtain a similar alignment among hearts and ease the visualization of the data, the long axis (LA) of the LV were aligned to the z-axis of each volume using rigid transformation. The LV was subdivided into regions using a polar coordinate system and the helix angles (HA) were calculated for each sample.

#### Retrospective sampling to the standard in-vivo resolution

The diffusion tensor images were retrospectively downsampled to two standard in-vivo resolutions of 2.0 × 2.0 × 5.0 mm^3^ and 2.5 × 2.5 × 8 mm^3^. The downsampling was performed in the log-Euclidean space. Diffusion tensor maps were computed as described in the previous section in the previous paragraph.

### Estimation of the whole heart label

A radio-frequency (RF) inhomogeneity field correction was applied to non-diffusion weighted images (b0) with ITK-N4 [23] to reduce the intensity bias and signal drop-off in the apical and basal regions associated with the RF coil. A binary mask was created and applied on both anatomical and DW images to segment myocardial tissue from regions with hyperintense signal corresponding to residual formaldehyde. Low and high cutoff thresholds were applied on the FA, trace, and DW images to define the binary mask as described previously in [19].

### Tractography processing

Tractography was used for visualization of global cardiomyocytes arrangement. Streamlines were generated with MRtrix3 [22] using the principal eigenvector of the diffusion tensor. Unless specified, the tractography parameters were defined as an FA-stopping threshold of 0.1, a maximum angle between steps of 45, a step size of 0.05 mm, a maximum length of 20 mm, and a minimum length value of 1 mm. Thus, streamlines terminated if a streamline made a sharp turn (angles larger than 45°), crossed a voxel with an FA of less than 0.1, extended to 20 mm, or extended outside of the masked region.

### Delineation of the aggregate cardiomyocytes bundle

**Fig. 1:**
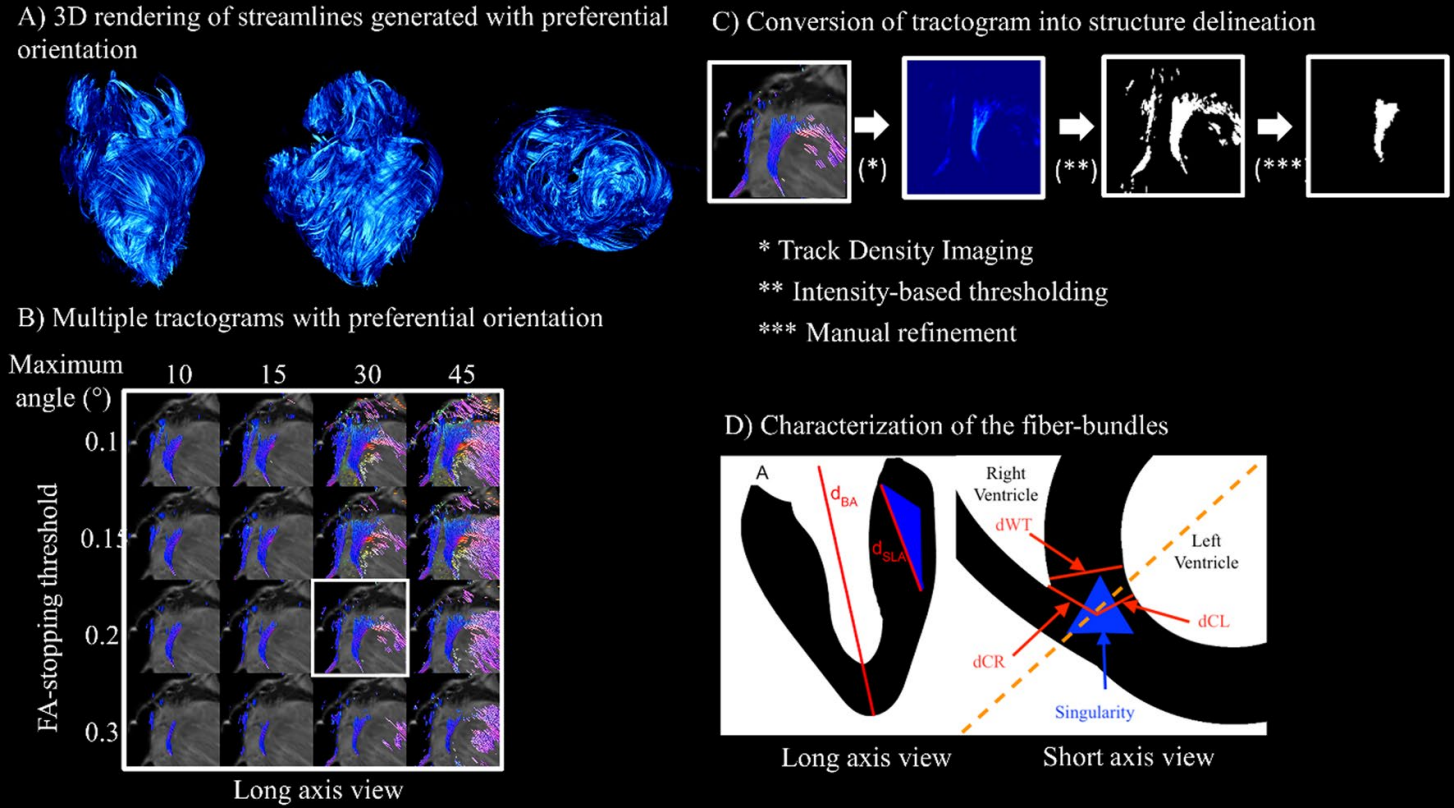
Summary of the tractography processing for delimiting and measuring the aggregate cardiomyocytes bundles located at the RVIP and also called singularity. (Left) **A** 3D rendering of streamlines filtered with preferential orientation. **B** Tractograms generation with different FA stopping thresholds and maximum angles. (Right) **C** Conversion of tractogram into structure delineation using track density imaging and intensity-base thresholding. **D** Schematics of the heart in long and short axes with all distances measurements and locations

Tractography processing (summarized in Fig. 1) was adapted to delimit and further quantify the size of aggregate cardiomyocytes bundles located at the RVIP, also referred to as the singularity.

i) Tractograms generation with preferential orientation.

As the aggregate cardiomyocytes bundle was visualized with a preferential orientation to the LA, tractograms were generated with a preferential orientation in the LA direction to isolate the aggregate cardiomyocytes bundle from the surrounding cardiomyocytes having a circumferential orientation (Fig. 1A). 16 tractrograms were generated by varying FA-stopping threshold and maximum angle as described in Fig. 1B. One set of parameters was picked arbitrarily (FA-stopping threshold of 0.2 and a maximum angle of 30°). The same values were used for all samples.

ii) Conversion of tractogram into structure delineation.

Track Density Imaging (TDI) was used to interpolate streamline information in a Cartesian frame [24]. Thus, for each element of a grid, the total number of streamlines was counted and converted into a scalar volume image. Then, intensity-based thresholding was applied on the scalar volume image to obtain a mask, delineating the aggregate cardiomyocytes in the heart with a preferential orientation to LA as shown in Fig. 1C. Lastly, refinement of the mask was manually performed using 3DSlicer (https://www.slicer.org/) to remove voxels outside the region of interest.

iii) Characterization of the aggregate cardiomyocytes bundles.

The volumes of the aggregate cardiomyocytes bundle and myocardium of the whole heart (without fat) were calculated by counting the corresponding number of voxels. The distribution of FA in the aggregate cardiomyocytes bundle and the whole heart were compared.

To quantify the size of the aggregate cardiomyocytes bundle compared to the whole heart, different distances have been used. These distances were measured manually using the software 3DSlicer and are described in. Figure 1D. The first (d_BA_) was the distance between the lowest point of the heart (Apex) and the highest point between the mitral valve, the same height of the top of the septum (Base). d_CR_ and d_CL_ were two distances between the center or the singularity and the closest point of the RV and LV respectively. The wall thickness (d_WT_) was calculated with the two points of the LV and RV chosen for d_CR_ and d_CL_. The d_SLA_ was the distance of the singularity on the LA view.

### STI estimation and tractography on microCT

3D STI was used on microCT images to estimate the aggregate cardiomyocytes orientation similarly to [14, 25]. First, the outer product of the intensity gradient vectors was computed to estimate the derivatives. The derivatives were then convolved with a Gaussian kernel with standard deviation σ = 6 and assembled to generate a symmetric second-moment matrix or structure tensor per voxel. The principal directions of the structure tensor in each voxel were extracted using eigen analysis. The third eigenvector (smallest magnitude eigenvalue) corresponded to cardiomyocytes orientation and was used to generate the streamlines. The tractography parameters were adapted to the signal-to-noise ratio and imaging resolution of the microCT images with an FA-stopping threshold of 0.1, a maximum angle between steps of 15, a step size of 0.05 mm, a maximum length of 20 mm, and a minimum length value of 0.5 mm.

### Histology

The sheep heart #1 was dissected to assess the presence of cardiomyocytes in the region depicting a triangular shape. The sample was transected from base to apex in large slices of 1 cm thick. The anterior part and the RV wall were removed to isolate the posterior wall of the heart. Sample was first rinsed in phosphate-buffered saline, then agitated at 4 °C for 24 h. After samples have been dehydrated, it was embedded in paraffin and sectioned at 6 µm in the transmural direction (or SA view). Tissue sections were stained with Masson’s trichrome for structural identification: collagen fibers in tissues were green; nuclei were black; and myocytes were red/pink. Slices were examined at 10× magnification on a tissue slide scanner (Axio Scan Z1, Carl Zeiss SAS, Marly le Roi, France). Images were visualized using Zen lite software (ZEN Blue 2.6, Carl Zeiss Microscopy, Thornwood, New York, USA).

### Electrophysiology simulation

**Fig. 2:**
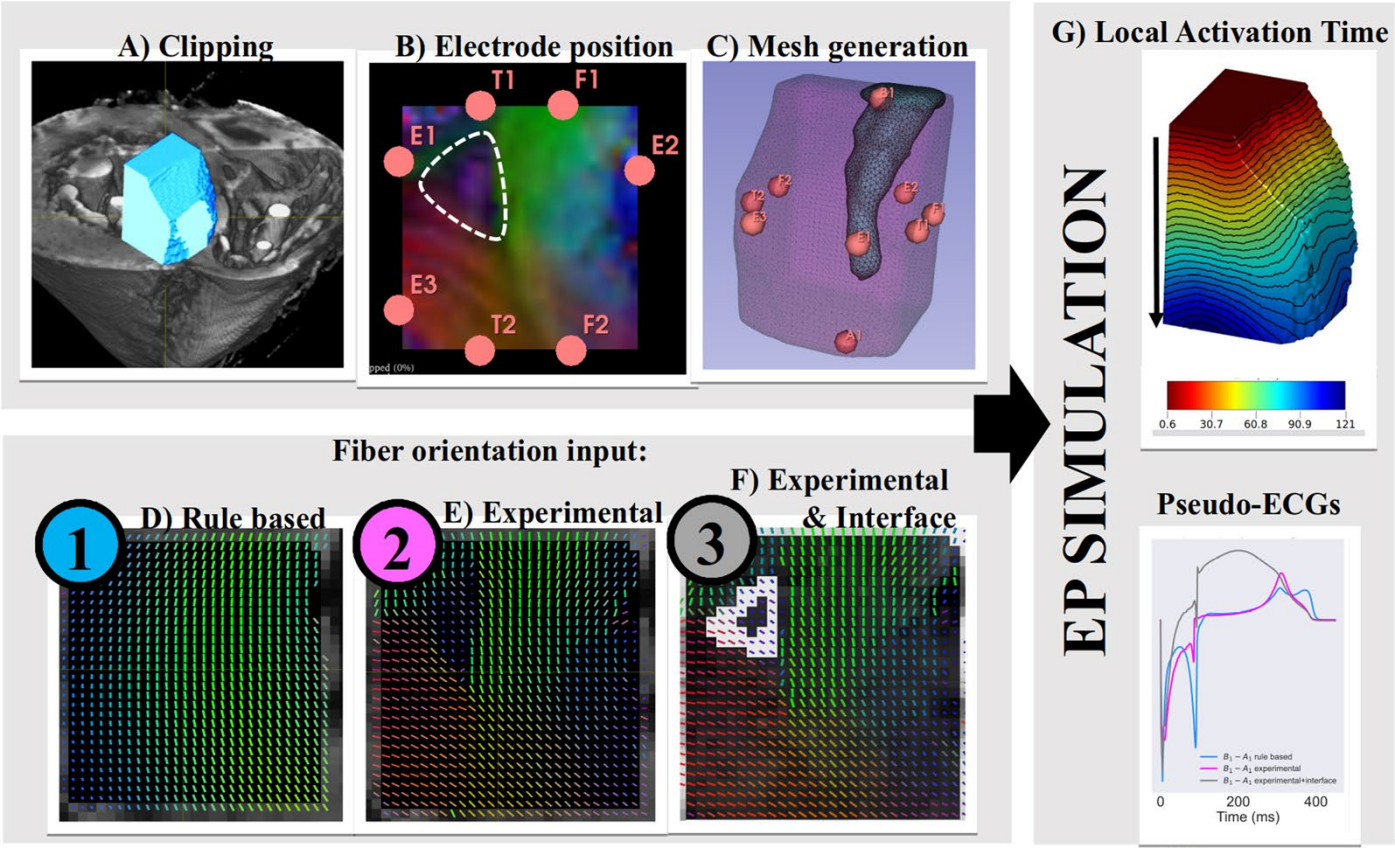
Workflow of the EP modelling study. **A** 3D rendering view of the sample #H1 with the superimpose region of interest used in the EP modeling. **B** Transverse view with electrode positions and experimental cFA maps that encode the x–y–z coordinates of the aggregate cardiomyocytes orientation. A white dashed contour highlights the identified aggregate cardiomyocytes bundles. **C** 3D rendering displaying the corresponding meshes and electrode positions. **D** Usual rule-based fiber configuration used as control in our studies. **E** Aggregate cardiomyocytes orientation derived from experimental DTI measurement. **F** Aggregate cardiomyocytes orientation derived from experimental DTI measurement plus a low conductivity interface at the border of identified aggregate cardiomyocytes bundles. **G** Activation time maps and unipolar signals and pseudo ECGs are computed as outputs of the computational model

Details about the simulations of the electrical activation propagation are provided in Fig. 2 and in Methods in the Additional file 1.

## Results

### Visualization of the aggregate cardiomyocytes bundle in the BIS wall in sheep hearts

**Fig. 3:**
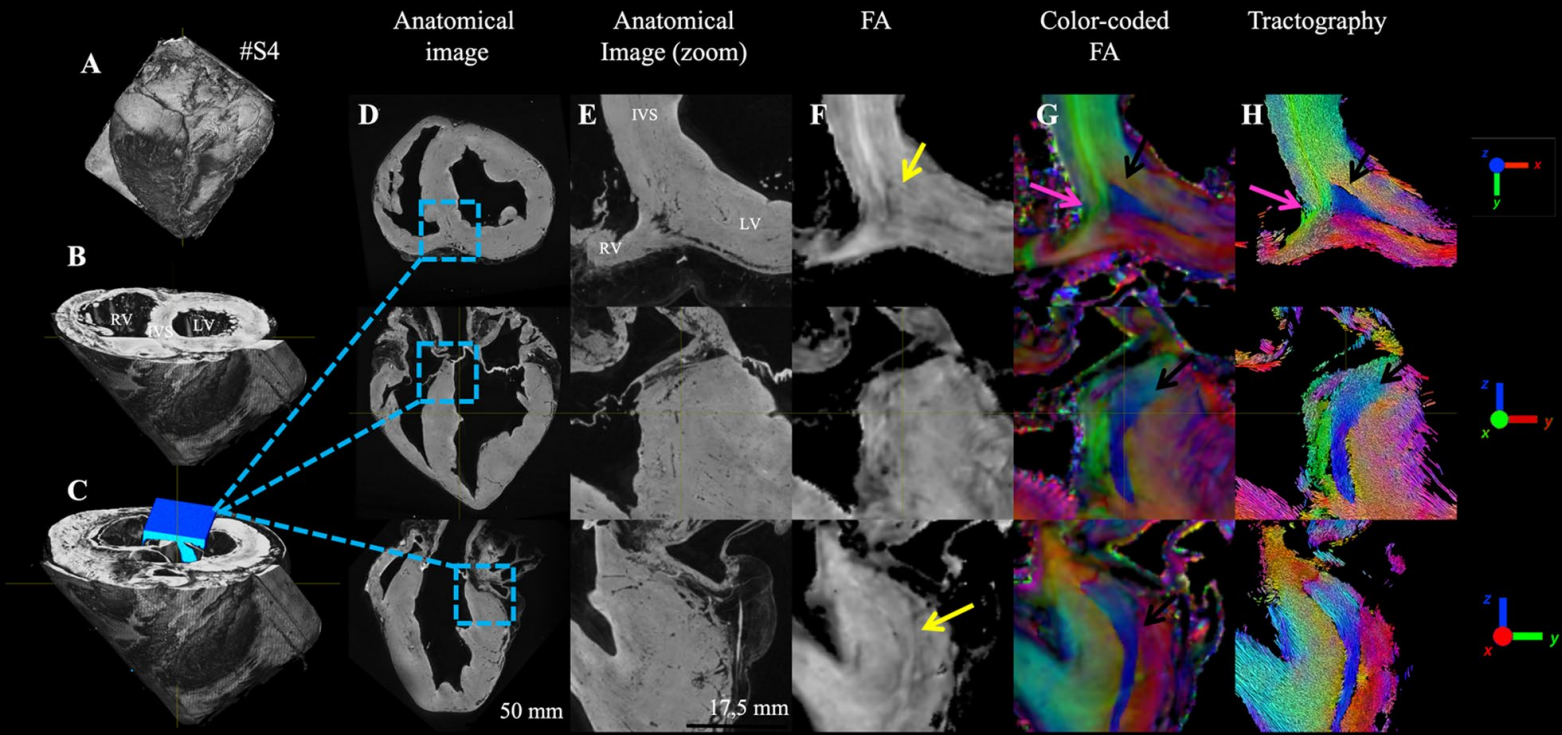
Visualization in ex-vivo sheep heart of the basal inferoseptal (BIS) left ventricular myocardium using anatomical images and diffusion tensor maps. (Left) A 3D rendering view of the sample #S4 displaying the whole heart (**A**), after slicing at the base level (**B**), with a blue cube showing the location of the inferior RVIP (**C**). (Right) Large views (**D**) and zoom views (**E**) of the anatomical images are shown with orthogonal views. FA maps (**F**). The cFA maps (**G**) encode the x–y–z coordinates of the aggregate cardiomyocytes direction derived from the DW images using a color code. Streamlines with the color-coding of the cFA (**H**)

Figure 3 displays a whole sheep heart #4 (A) and the location (blue cube) of the BIS region using 3D rendering (B, C). Anatomical data shows a relatively homogeneous tissue (D, E). The FA maps computed from the diffusion tensor images (F) show a more heterogeneous distribution. A triangular pattern presenting lower FA values on the edge is somehow visible (yellow arrows). The triangular pattern has a clear image signature on cFA or tractography map (indicated by pink arrows) with the aggregate cardiomyocytes oriented in base to apex direction starting at the inferobasal crux and ending at the middle of the inferoseptal wall of the LV. The surrounding aggregate cardiomyocytes have, in the endocardial part of the LV, a standard circumferential orientation. On the opposite side, at the RVIP, a corridor of aggregate cardiomyocytes, indicated by a purple arrow in the SA view, goes from the septum to connect with the tissues of the RV wall.

Additional file 1: Fig. S1 plots the profile of the FA map with the corresponding SA view at the location of the identified pattern. The mean value of FA along the line is: 0.20 ± 0.08 and a decreased value is noticeable in the edge of the pattern indicating potential aggregate cardiomyocytes discontinuity or crossing in this region.

**Fig. 4:**
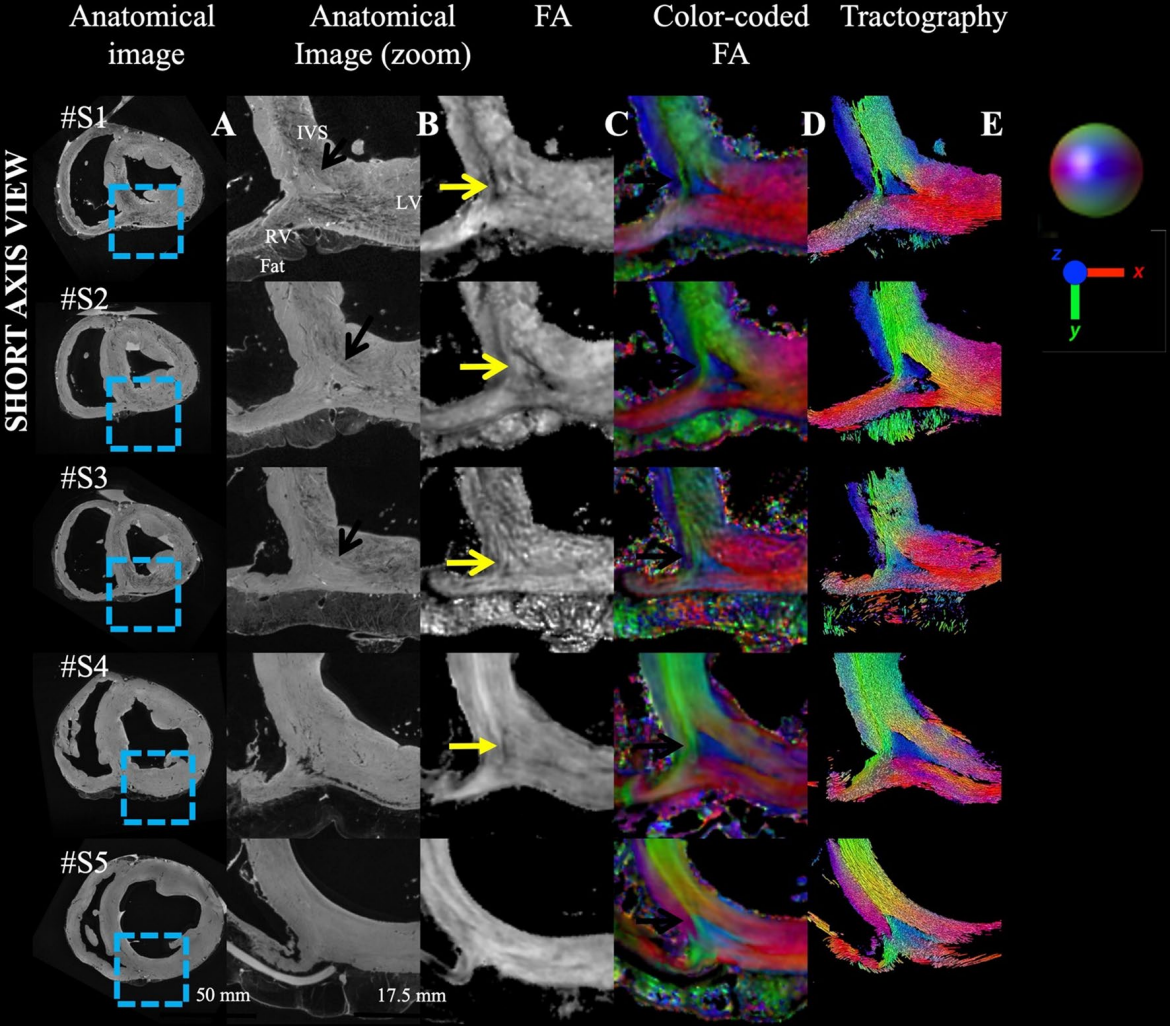
Comparison across ex-vivo sheep hearts (N = 5) of the aggregate cardiomyocytes orientation in the BIS left ventricular myocardium in SA view using anatomical and diffusion tensor images. The legend of the metrics of Fig. 3 applies

Figure 4 investigates the reproducibility across different samples of the same species in five sheep hearts. In anatomical images, the intensity is relatively homogeneous except for a few areas with reduced signal in the epicardial fat. Nevertheless, small variation in gray contrast in samples #S1–S3 are indicated by black arrows. #S1–S4 (fixed in a pseudo-systolic phase) show a large wall thickness, the last sample #S5 (fixed in a pseudo-diastolic phase) presents a small wall thickness. On hearts #S1–S4, the same decrease in the FA value is visible (indicated by the yellow arrow) on the edge of the triangular pattern, while it is not visible on sample #S5. Smooth transition of aggregate cardiomyocytes orientation is visible (gradient of green to purple to red) indicating a radial (in-plane) aggregate cardiomyocytes orientation in the endocardial part of the LV while aggregate cardiomyocytes orientation changes abruptly between adjacent voxels in the interventricular septum (#S1–S3, #S5) and in the middle of the RVIP for all samples with a blue color that depicts an apex-base orientation (#S1–S5). The contrast of the color, slightly different in sample #S5, indicates a difference in orientation, but similar discontinuities in aggregate cardiomyocytes orientation remain. The LA view is available in Additional file 1: Fig. S2 that displays the extension of the aggregate cardiomyocytes bundles that vanished close to the mid-ventricular location. Additional file 1: Fig. S3 displays both SA and LA view with HA maps.

**Fig. 5:**
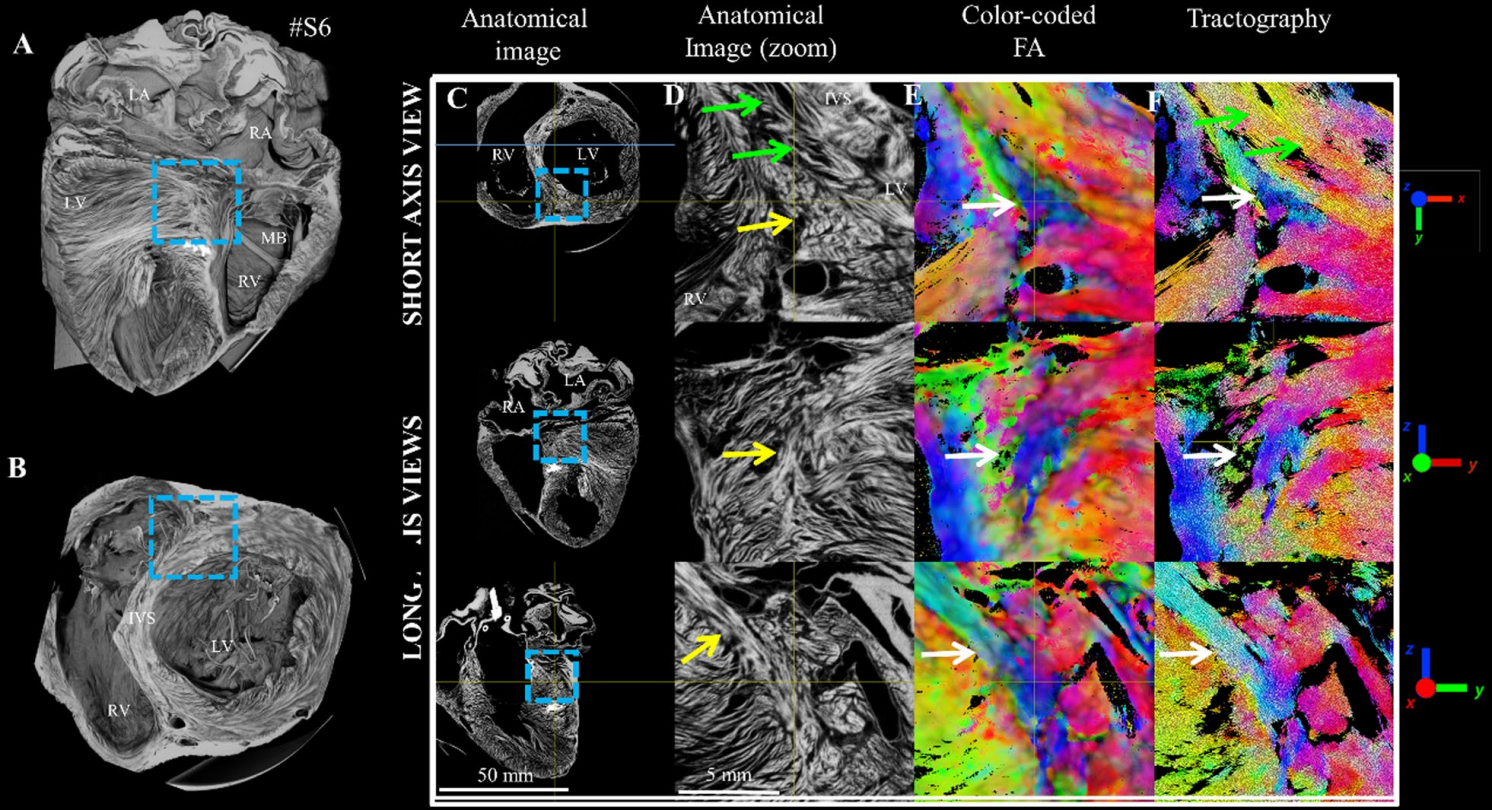
Visualization of the BIS left ventricular myocardium using microCT images and structure tensor derived maps in ex-vivo sheep heart. (Right) A 3D rendering view of the sample #S6 displays the whole heart (**A**), after slicing at the base with a blue dotted cube showing the ROI in the inferior RVIP (**B**). Anatomical images (**C**, **D**). cFA map (**E**) and tractography map (**F**). Circumferential fibers (green arrow); aggregate cardiomyocytes bundles with base to apex fiber orientation (yellow arrows)

Figure 5 displays the cardiomyocytes architecture using microCT and STI. The left panel displays the whole sheep heart #S6 (A) using 3D rendering and the location of the BIS area using a blue cube (B). On the right panel, the anatomical contrast offers a direct visualization of the laminar structure. In the SA view, circumferential cardiomyocytes are visible (green arrow) while cardiomyocytes crossing the view are located in the middle of the inferior RVIP (yellow arrows). The longitudinal views also show the presence of cardiomyocytes bundles with specific base to apex orientation (yellow arrows). Visually, the cardiomyocytes’ density appears more pronounced in comparison to the surrounding cleavage planes showing alternating cardiomyocytes and air layers. The reconstructed cFA maps and the streamlines display a triangular pattern with cardiomyocytes bundles in base to apex orientation (white arrows).

**Fig. 6:**
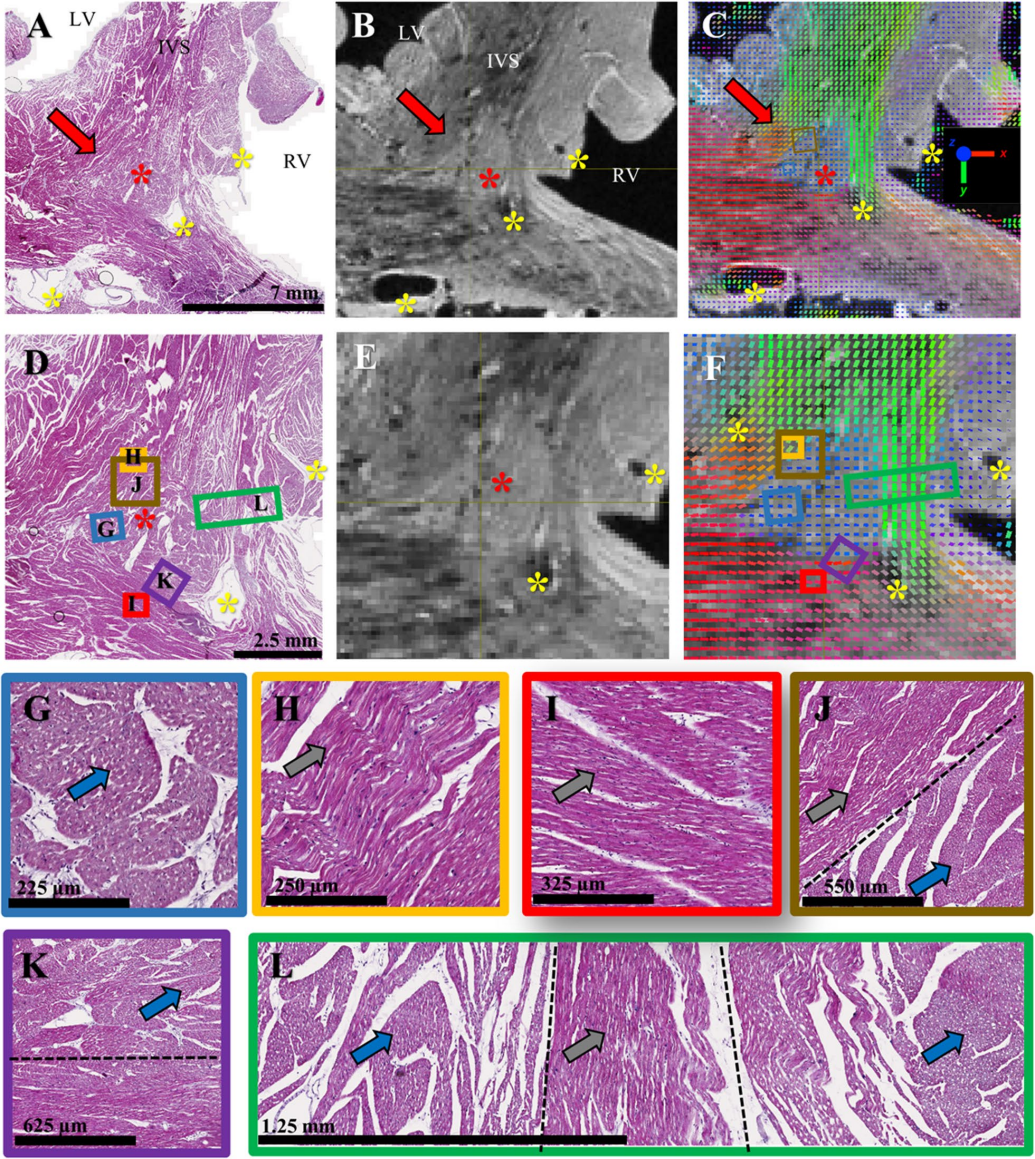
Histological examination of the basal inferoseptal (BIS) left ventricular myocardium. Histological slice (**A**) stained with Masson’s trichrome and corresponding anatomical image (**B**) and cFA maps (**C**) in the basal inferoseptal (BIS) left ventricular myocardium. Anatomical landmarks such as vessels having a black contrast are indicated with yellow asterisks. The red arrow and red asterisk indicate the location of the region depicting a triangular shape. Panels **D**–**F** show an enlarged view of the previous panels. Zoomed rectangle regions (**G**–**L**) are indicated and are located either inside the region (**G**), outside (**H**, **I**) or at the junction (**J**–**L**). The blue and gray arrows indicate cardiomyocytes in transverse and longitudinal direction respectively. The dash black lines indicate roughly the location of discontinuities in myocyte orientation

Macroscopic examination and histological examination were performed on sheep heart #S1. Additional file 1: Fig. S4 shows a photograph of the sample after short-axis transection, confirming the macroscopic size of the identified region. Visual agreement with the anatomical MRI image is evident. Figure 6 investigates the presence and orientation of cardiomyocytes in the region. The histology slice was first compared to MRI-derived data using landmarks (yellow asterisks) at the vessel locations (Panels 6A–F). Tissue staining revealed the presence of cardiomyocytes in the entire triangular regions and a clear image signature about myocyte orientation was found. Inside the region (panel 6G), myocytes are in transverse orientation depicting a base-apex direction. The surrounding myocytes outside the region (panel 6H and 6I), have a standard longitudinal orientation. Lastly, visualization of abrupt mid-myocardial change in myocytes orientation is clearly visible (Panels 6J–L) at the junction confirming the cardiac structure discontinuities revealed by DTI measurements.

### Visualization of the aggregate cardiomyocytes bundles in the BIS area in human hearts

**Fig. 7:**
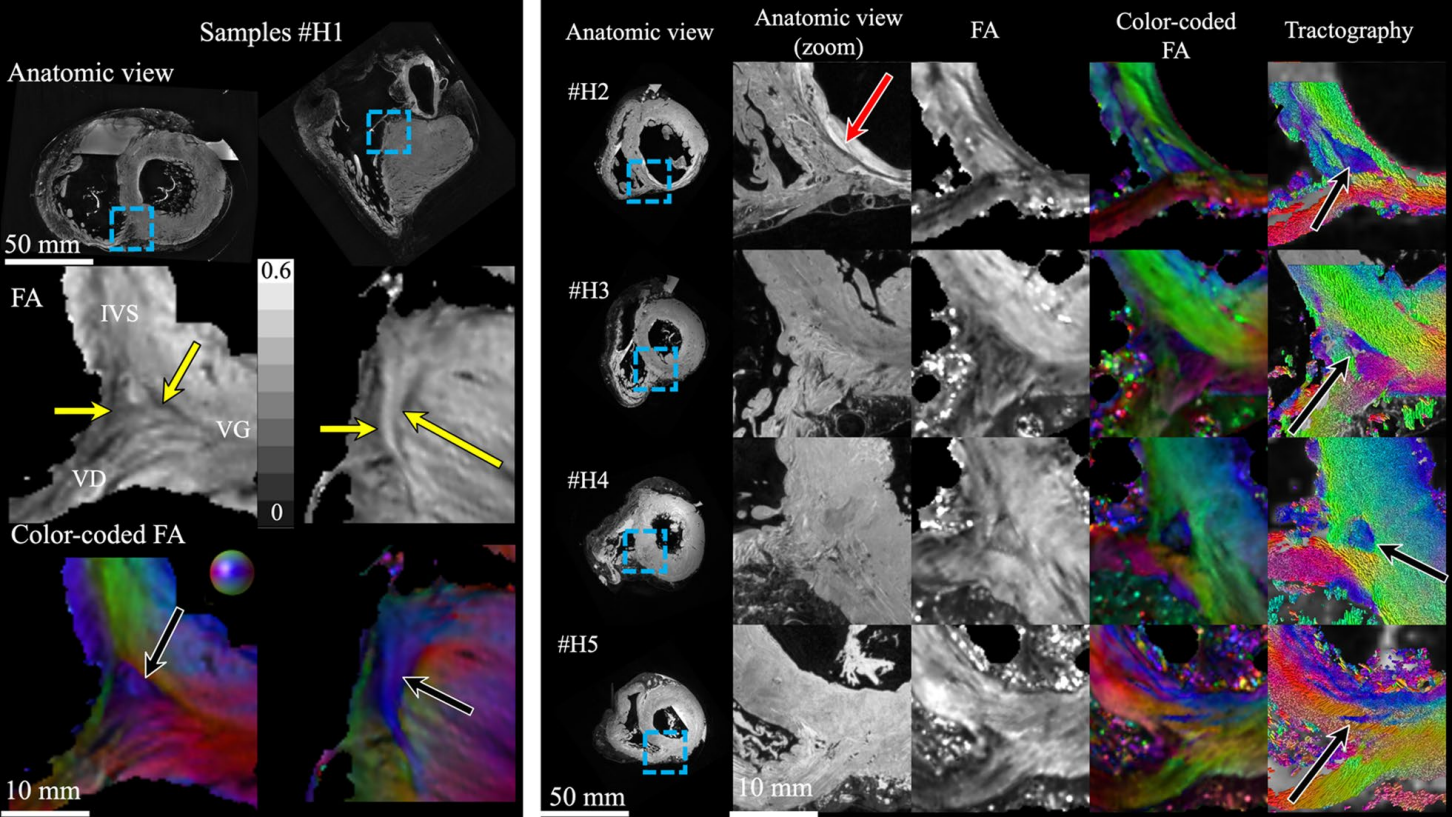
Comparison across ex-vivo human hearts (N = 5) of the aggregate cardiomyocytes orientation in the BIS left ventricular myocardium in SA view using anatomical and diffusion tensor images. Right panel: sample #H1; Left Panel: samples #H2-5. The legend of the metrics of Fig. 3 applies. Black arrows indicate the "fiber" bundle in the RVIP. Red arrow indicates myocardial scar

**Fig. 8:**
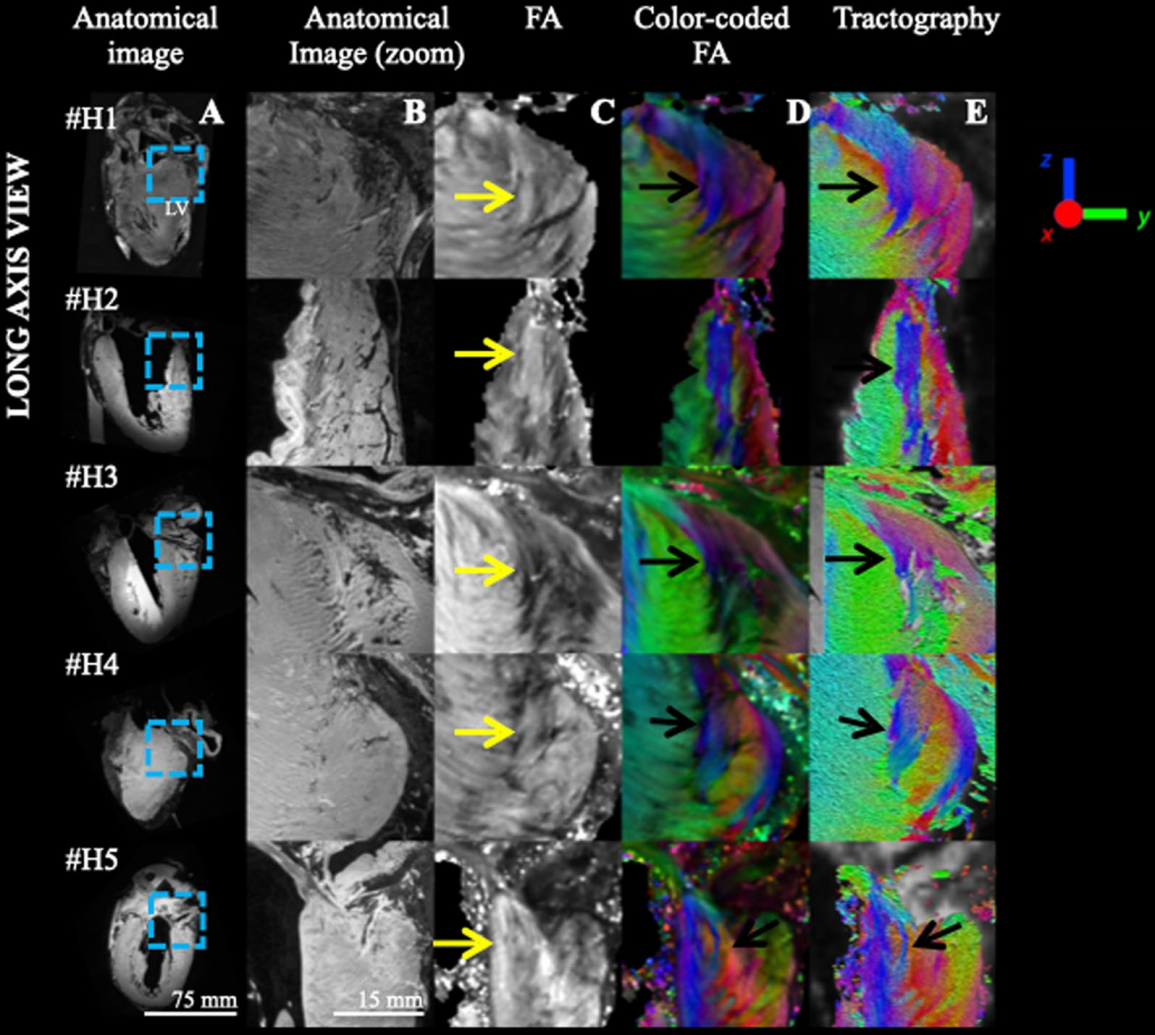
Comparison across ex-vivo human hearts (N = 5) of the aggregate cardiomyocytes orientation in the BIS left ventricular myocardium in LA view. The legend of the metrics of Fig. 3 applies

Figure 7 investigates the presence and the reproducibility across different human samples. Samples #H1–2 presented myocardial infarction scars. Samples #H3 and #H4 came from patients with minimal cardiac disease and #H5 came from a healthy, albeit aged, subject. Gross morphology as well as myocardial viability was highly heterogeneous. A large infarct scar is visible on sample #H2 with reduced wall thickness on the inferior and inferolateral walls. The presence of epicardial fat is noticeable in all samples. Anatomical data shows a relatively homogeneous tissue with the exception of sample #H2. On the FA map, the edge of the triangular pattern is well visible for #H1, #H2, and #H4 samples (yellow arrows) in the SA view, while it is not clearly visible on sample #H3 and #H5. The cFA maps and the streamline rendering display the typical pattern in each sample with aggregate cardiomyocytes bundles in base to apex orientation indicated by the white arrow. Although it is up to the reader's appreciation, the geometry remains triangular for samples #H1–4 but with a larger heterogeneity. The sample #H5 presents relatively small aggregate cardiomyocytes bundles at the junction and the pattern does not look triangular. The LA view is available in Fig. 8, and shows the singularity dives from base to apex at a depth of ~ 2 cm. Additional file 1: Fig. S5 displays both SA and LA view with helix angle maps.

### Characterization of the fractional anisotropy in the singularity

**Fig. 9:**
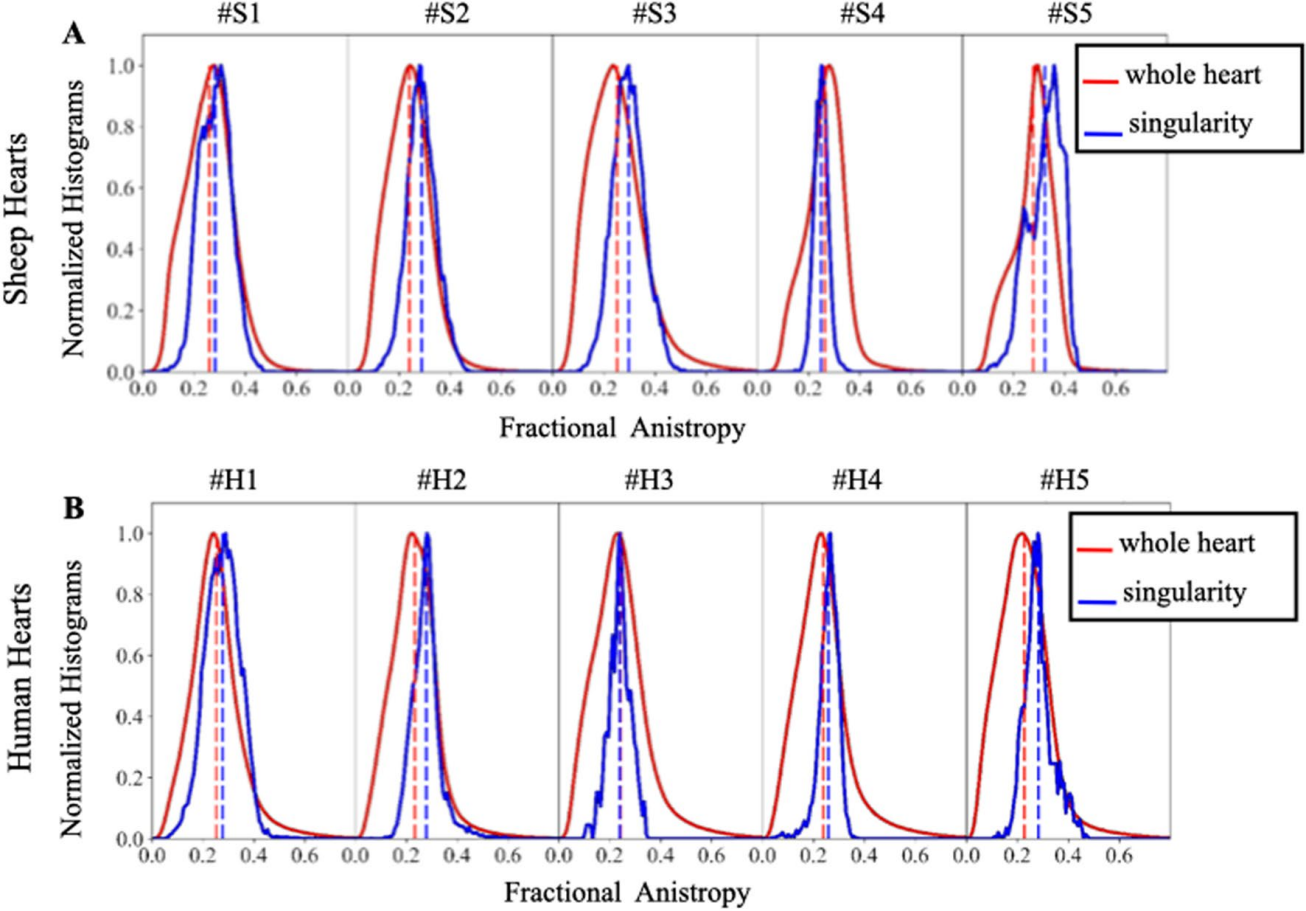
Normalized histograms of FA for sheep (**A**) and human (**B**). The lines represent the distribution of FA in the whole heart (red) and the distribution of FA in the singularity (blue). Dashes lines indicated the average value

Figure 9 shows the distribution of FA in the singularity (using roughly 15,000 voxels) and the whole heart (using roughly 5.10^6^ voxels) for all samples. In average, there is a small shift between the FA distribution in the whole heart and in the singularity for both sheep (except #S4) and human samples (except #H3). The average mean and standard deviation values (summarized in Additional file 1: Table S1) in the whole heart are (mean ± std: #S: 0.26 ± 0.09, #H: 0.24 ± 0.10) and in the singularity (mean ± std: #S 0.29 ± 0.06, #H: 0.27 ± 0.05) for sheep and human samples respectively. The histogram distribution of the FA in the singularity is relatively homogeneous across samples and species, and a narrow distribution of FA was found in comparison to the whole heart distribution.

### Sizing of the singularity and potential of in vivo imaging

Additional file 1: Fig. S6 and Table S2 summarize the size and volume estimation of the aggregate cardiomyocytes bundles after structure delineation using TDI. Base-Apex distances (dBA) of the ventricle ranged from 70 to 85 mm for both species. Wall thickness (dWT) was 15 mm on average and showed higher variability for human hearts. The long axis of the singularity (dSLA) was estimated to 23.0 ± 5.6 mm (both for human and sheep hearts) with the exception of the sample #H5. The singularity is closer to the RV cavity than the LV cavity as the wall thickness of the LV is higher. In all cases, the singularity is mostly inside the LV assuming that the RV/LV limit is a B-slice interpolation of the LV epicardium. The volume of the singularity ranges from 0.1 to 0.6 cm^3^ with a mean value of 0.35 ± 0.15 cm^3^. Additional file 1: Fig. S7 shows the cFA maps of all human samples downsampled to 2.0 × 2.0 × 5 mm^3^ and 2.5 × 2.5 × 8 mm^3^ and display a few remaining voxels of the singularity. Additional file 1: Fig. S8 shows the RVIP of #S2 with different b-values. The first row shows the average of DWI volumes and the second shows the cFA as a function of b-values. The cardiomyocyte orientation starts to be visible from b = 350 s/mm^2^ and the aggregate of myocytes in base apex orientation seems more visible from b = 500 s/mm^2^ and display a similar pattern like in Fig. 4.

### Cardiac electrophysiology simulation

**Fig. 10:**
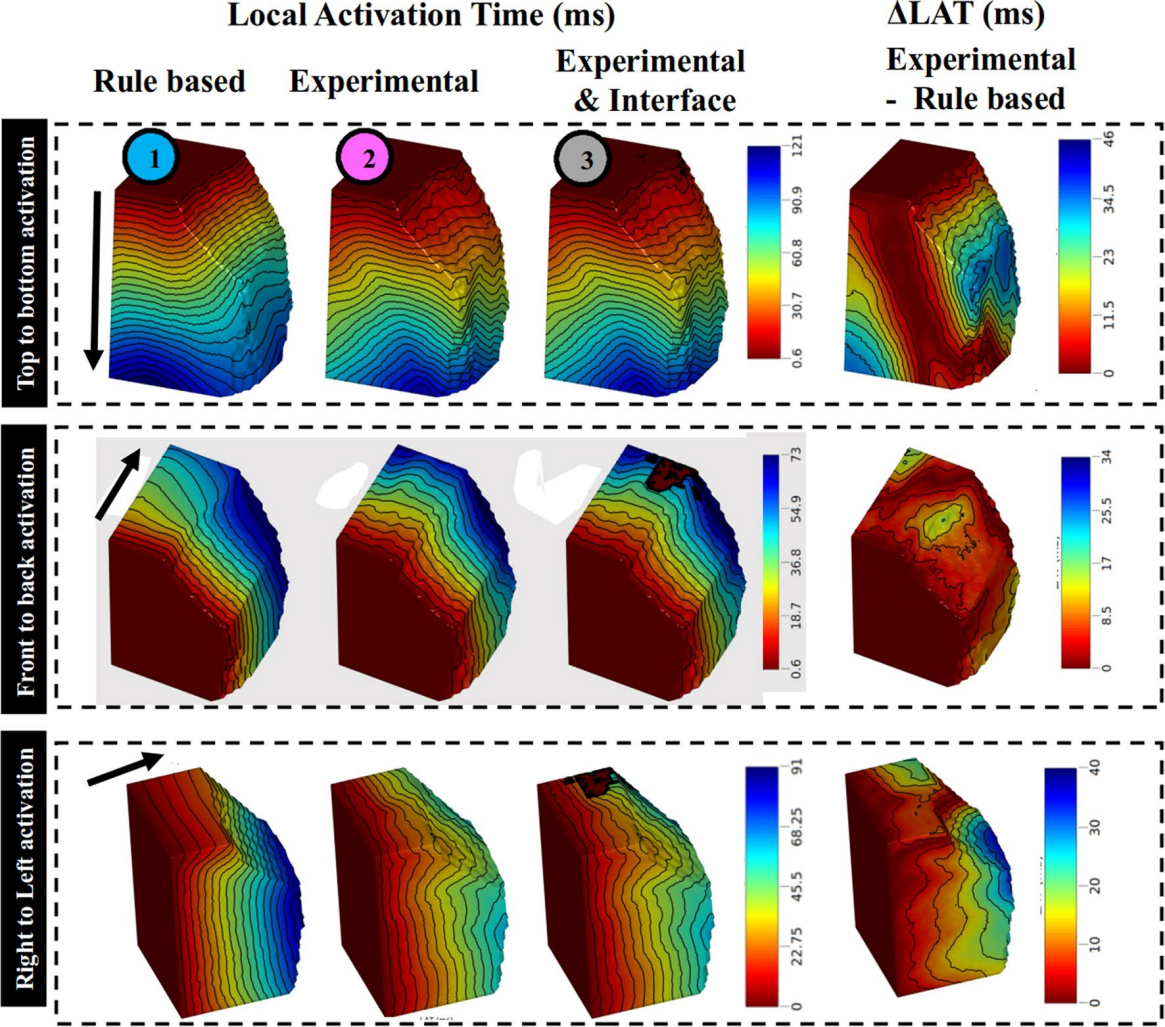
Activation time maps (left) and difference in activation time maps (right) for the different cases considered in our study. Usual case with rule-based fiber orientation (1st column). Cases incorporating aggregate cardiomyocytes orientation derived from experimental DTI measurement (2nd column) and with the addition of a non-conducting interface at the border of the identified region (3rd column). The activation was initiated by surface pacing as indicated by the black arrow for three different locations

Spatial distribution of local activation times is shown in Fig. 10 using the usual rule-based "fiber" orientation (1), experimentally measuremented “fibers” (2), and with a non-conducting interface at the region border (3). For all activation patterns, incorporating experimental “fibers” slows wavefront propagation, leading to significant delays ranging from 10 to 40 ms. Such differences are reflected in LAT maps. The average LAT differences between cases (1) and (2) are 21.4  ± 8.0 ms, 19.9  ± 6.6 ms and 17.2  ± 4.8 ms for the top to bottom, left to right and front to back propagations, respectively. LAT differences between cases (1) and (3) are 22.0  ± 9.8 ms, 21.3  ± 9.7 ms and 19.0  ± 9.0 ms as before.

“Fiber” orientation influence is reflected in the pseudo-ECGs (Additional file 1: Fig. S4). Only electrode pairs perpendicular to the propagation wavefronts are shown. Incorporating experimental “fibers” changed the morphology and amplitudes of all pseudo-ECGs. For top-to-bottom stimulation, the highest amplitude of the B1-A1 voltage changes from 4.96 mV to 4.8 mV to 5.68 mV, for rule-based, experimental, and experimental + interface, respectively. Analogously, for front-to-back activation, T1-T2 voltage amplitude changed from 7.69 to 4.05 and 3.45 mV, for rule-based, experimental, and experimental + interface, respectively. Finally, for left-to-right activation, the E1-E2 electrode voltage amplitude changed from 1.97 mV to 4.85 and 4.58 mV, as previously.

The non-conducting interface is more apparent for bipolar points crossing it. The amplitude discrepancies between test cases (2) and (3) are more pronounced for top to bottom activation and right to left activation.

The RVIP bundle interface effect is clearly seen in electrograms traversing it. Current flow is affected by two factors: (1) the interface is non-conducting with holes which slow down propagation, since the bundle is excited at discrete points, rather than a plane wave, and (2) fiber direction within the RVIP bundle is distinct from adjacent myocardium, producing different conduction velocities within the bundle relative to the electrodes. Also, experimentally measured fibers result in faster trans-slab conduction to change the time of the second spike in the activation complex, corresponding to far surface excitation. This peak was the same for experimental fibers regardless of interface, but different for rule-based fibers. The interface is responsible for the pseudo-ECG morphology. When electrical coupling across an interface depolarizes transmembrane voltage, but insufficiently to initiate excitation, ST elevation results [26]. When electrode pairs do not cross the interface (F1-F2 & E2-E3), pseudo-ECGs are almost equal.

## Discussion

In this study, the microstructure organization of two large mammalian species was investigated in the BIS myocardium using three distinct imaging methods. First, we highlight the existence of abrupt mid-myocardial cardiomyocyte orientation changes using DTI at an isotropic spatial resolution of 600 µm, delimiting a triangle-shaped region, in both sheep and human samples. The discontinuity can be identified either using the FA maps that quantify the anisotropy of water molecule motion in a voxel, or using directional information of the diffusion tensor via cFA maps or streamlines representation. FA distribution was found systematically equal or higher in both species in the identified aggregate cardiomyocytes bundles than in the whole hearts. The mean FA found in the whole hearts was in agreement with previous ex-vivo studies [27]. The proposed description was then confirmed by microCT acquisition that offers direct visualization of the laminar structure in this region.

In sheep hearts, the triangular pattern shows comparable configurations across samples corresponding to the systolic phase. The sample fixed in the diastolic phase reports an overall shift in aggregate cardiomyocytes orientation but the discontinuity remains clearly visible. In human hearts, a more heterogeneous pattern is noticeable in particular for the sample #H2 which has an important scar along the free wall (Fig. 7) but is visible in all samples. We quantified the volume and size of the aggregate cardiomyocyte bundles using state of art tractographic processing. The segmentation of the singularity was performed using TDI, which allows the delineation of specific regions by converting streamline statistics in scalar images [24]. The mean length of dSLA and volume of the aggregate cardiomyocytes were 23.7 ± 4.1 mm and 0.42 ± 0.10 cm^3^ for sheep, and 20.8 ± 7.0 mm and 0.26 ± 0.15 cm^3^ for human hearts, respectively. To assess the presence of cardiomyocytes in the region depicting a triangular shape, macroscopic examination and histological examination were performed on sheep heart #S1. The histological results (Fig. 6) confirm the presence of cardiomyocytes in the region and demonstrated an excellent match between DTI and histological technique to follow the main cardiomyocytes orientation. Lastly, the existence of cardiac structure discontinuities (or abrupt change in cardiomyocytes orientation) revealed by DTI measurements was confirmed at the junction of the identified region. Our study is proof of existence, but generalization of our observations is difficult regarding the low number of samples (N = 11) and several questions remain open. The findings are at odds with literature that reports either highly organized myocyte architecture or disorganized myocyte architecture at the RVIP. Indeed, a recent meta-analysis on ventricular cardiomyocytes [28] refutes the presence of internal tissue structures that could be interpreted as boundaries.

### The physiological hypothesis

The presence of these aggregate cardiomyocytes bundles in healthy and relatively young animals could suggest that this aggregate cardiomyocyte bundles organization is physiological. A naive and speculative hypothesis attributes the presence of this structure for stiffening the region located at the intersection of the ventricular and atrial chambers. A similar role has been reported for the papillary muscles [29]. While different configurations and sizes are reported in the study, the inclusion of ex-vivo human samples could not confirm this hypothesis as most donors had a history of heart disease. In such a context, the human sample #H2 that presents a large scar in the inferior and inferolateral wall is an interesting case. This heart had undergone a significant reduction of the myocardial wall thickness in the LV, and a massive replacement of the working myocardium by fibrosis, visible on the anatomical images (Fig. 6). However, we can distinguish on the FA map, an isolated region with a higher value of FA where the aggregate cardiomyocyte bundles are present in base to apex direction. This could be interpreted that the region is stiffer and less sensitive to remodeling. Additionally, rare examples are also present in the literature such as Fig. 2 from [30], that showed a similar pattern in a fetal heart after 20 weeks of gestation or in canine heart [31]. Prior work by Mekkaoui et al. [15] used tractographic techniques for myocardial structural characterization and showed the presence of discontinuity at the insertion point. The propagation angle, a tractography-based metric quantifying the curvature of streamlines, computed in this study was consistently found higher at the RVIP. While under debate in the literature, we can also underline that gross pathology description (limited to the content of 3.2 paragraph: “The intervetricular sulci”) by Kocica et al. [32] closely match our current and previous [16] experimental findings.

### The cardiac remodeling hypothesis

The cardiomyocyte orientation at the RVIP follows the rule-based helix angle orientation in the literature, either in microstructural or modeling studies [33]. Nevertheless, it is often accepted that the cardiomyocyte organization is much more complex due to the intersection of the ventricular cavities. Stretch-induced adaptive reorientation of cardiomyocytes has been reported in the literature [34] and is supposed to play a large role in the pathological process. Thus, cardiac remodeling could be considered sufficient to substantially alter the orientation in the LV of circumferential cardiomyocytes toward base to apex direction. The myocardium at the junction could be subjected to a complex mechanical pressure that might lead to heterogeneous shear force or stress. Nevertheless, such a hypothesis would imply an asymmetrical and localized response to the mechanical pressure. First, no evidence of similar aggregate cardiomyocyte bundles was found in the superior wall. Secondly, aggregate cardiomyocyte orientation changes rapidly and not gradually across the inferior RVIP. Computational mechanical models could be envisioned to explore different scenarios of remodeling but are out of the scope of the paper.

### Fibrosis implications

RVIP-LGE was reported in multiple patient populations including hypertrophic cardiomyopathy [6, 35], dilated cardiomyopathy (DCM) [5], non-ischemic cardiomyopathy (NICM) [7], in heart failure patients with preserved ejection fraction [8], in highly trained endurance athletes [9, 36], and in all patients with pulmonary arterial hypertension (PAH) [3, 13]. The clinical relevance of RVIP-LGE and prognostic differ in the literature [2, 37, 38]. The LGE pattern was found to be a nonspecific clinical marker in patients without cardiac damage [4] but also a marker of PH for more advanced disease [37]. The described aggregate cardiomyocyte bundles probably play a role in the aforementioned clinical studies. The presence of focal LGE could be a chronic mechanical stress marker.

Two mechanisms could be considered within and at the border of the aggregate cardiomyocytes bundles: i) in this region, the forces related to ventricular contraction could be expressed differently at the cell level of these two aggregate cardiomyocytes populations (circumferentially and apico-basal oriented) by producing a longitudinal and perpendicular stretch respectively which may result in cell remodeling, and ii) the presence of an abrupt change in orientation might also be a potential substrate for interstitial fibrosis. Experimentally, a decrease of FA was noticeable at the interface of the aggregate cardiomyocyte bundles. This could indicate either cross orientation of cardiomyocytes within a voxel, or also the presence of interstitial fibrosis [26].

### Electrophysiology implication

Adequate simulation of arrhythmic phenomena requires accurate patient-specific data. Although cardiomyocyte orientation has been shown to have major effects on wavefront propagation in electrophysiology modeling [39], most studies rely on rule-based information derived from ex-vivo canine samples [40] that do not fully reflect the heterogeneity of the human myoarchitecture. In this study a realistic cardiomyocyte orientation distribution was compared to the usual rule-based model in a block of tissue located in the BIS myocardium. Both spatial distribution of LAT and pseudo-ECGs were strongly impacted with delayed LATs and changes in amplitude and morphology.

The impact of fibrosis is usually driven via the in-silico integration of heterogeneous tissue composition at the cellular level [41]. Here we suggested the presence of cardiomyocyte orientation discontinuity as a potential substrate for fibrosis and leading to  conduction slowing. To investigate such a scenario, a simulation was performed with the presence of a non-conducting interface at the border of the identified region. The morphology of pseudo-ECGs was changed, specifically by adding the interface, a notch was observed in the bipolar signals in each of the activations. Reentry or electrical wavefront discontinuity leading to arrhythmia was not observed but were unlikely due to the small dimension of the block of tissue and the length of the action potential duration. Nevertheless, uncommon idiopathic ventricular arrhythmias (VA) [42–44] originating from the basal inferoseptal left ventricular myocardium have been reported and could originate from the abovementioned aggregate cardiomyocyte bundles.

### Limitations

The extension of these findings is, however, limited by a large number of factors with notably the small number of samples and the contractile state of the ex vivo samples. In vivo imaging of the identified structure could be envisioned using new DTI-CMR acquisition [45] but was out scope of the paper. To investigate such a possibility, the diffusion tensor of all human samples were downsampled up to 2.5 × 2.5 × 8 mm^3^. The new voxel size (50 mm^3^) is 231 times larger than the previous one (0.216 mm^3^) and increases the partial volume effect. Nevertheless, a few remaining voxels (< 10 per sample) displayed in Additional file 1: Fig. S7 still shows characteristic cardiomyocyte orientation in the RVIP in samples 1 to 4. However, the transition to in-vivo acquisition is extremely challenging due to the combined cardiac and respiratory motion. We also investigated the influence of b-values and found that a b-value b >  = 500 s/mm^2^ might be necessary to visualize the identified structure. It is reasonable to underline that the two main factors to image the region will be a sufficient spatial resolution in plane and in slice thickness to avoid too large a partial volume and a drop in FA, and of course adequate signal-to-noise ratio. The usual limitations regarding the interpretation of tractography and streamlines apply due to the large difference between the myocyte size and maximum streamline length.

In-vivo cardiac DTI could serve as a biomarker of structural remodeling and may be used in the close future for risk stratification. Relationships between FA, fibrosis and ventricular arrhythmia have recently been investigated in this way [46]. Such clinical perspectives are encouraging for improved diagnosis of cardiac pathologies but also underline the need for a precise description of the baseline cardiomyocyte organization at multiple scales and imaging modalities. To some extent, the concept of myocardial disarray must be used with caution, as the more complex aggregate cardiomyocyte orientation in the inferior RVIP may not be considered as pathological myoarchitectural disarray but might be a substrate for pathological mechanisms.

The development of high-resolution accelerated diffusion MRI acquisition coupled with standardized sample preparation and post-processing pipeline could help in designing a modern atlas of the cardiac aggregate cardiomyocyte architecture [15, 47, 48]. To support our findings and promote the reproducibility of the results, the raw data and diffusion tensor images used in the article and minimal scripts to reproduce the figure of the article were released (see data availability section).

## Conclusion

The study was the first to describe the 3D cardiomyocyte architecture of the BIS ventricular region in large mammalian hearts. A triangular region depicting a arrangement of cardiomyocytes in the basal–apical direction starting at the inferobasal crux and ending at the middle of the inferoseptal wall of the LV was found in both species using diffusion tensor imaging methods. The results were confirmed by microCT imaging and histology. EP computational models were then used to investigate whether the presence of cardiomyocytes orientation discontinuity, being a potential substrate for fibrosis, could promote a conduction slowing. Lastly, this peculiar arrangement may provide an explanation as to why this region may be damaged by increased right ventricular wall stress. It is hoped that these basic research findings could help in providing a better understanding of the biological processes underlying cardiac remodeling or arrhythmia vulnerability and will pave the way for linking microstructural observations to distinct clinical manifestations.

### Supplementary Information

**Additional file 1.** Supplementary figures and tables.

## Data Availability

Link to the data is indicated here (https://github.com/valeryozenne/Cardiac-Structure-Database/tree/master/Article-4). Results are visible with the mrview viewer using the panels overlay/tensor/tractography. Command scripts that compute in native space (diffusion tensor, first eigenvectors, tractography) and command-line instructions for generating figures are also available on the same webpage.
