# Supplementary material for: Cardiac structure discontinuities revealed by ex-vivo microstructural characterization. A focus on the basal inferoseptal left ventricle region
Source: J Cardiovasc Magn Reson. 2023 Dec 14;25:78. doi: 10.1186/s12968-023-00989-y (PMC10720182; doi:10.1186/s12968-023-00989-y)
Supplement: Supplementary file 1 — Additional file 1. Supplementary figures and tables. [file 12968_2023_989_MOESM1_ESM.docx]

**Supplementary Materials**

Methods:

EP Simulation: A 1.7 cm × 1.8 cm × 2.5 cm wedge, discretized at 350 μm along each dimension, yielding 285,000 nodes and 1.5 million tetrahedra, was constructed to represent the basal inferoseptal wall of the human heart #H1. We considered three scenarios (summarized in Fig. 2) for our studies in this geometry. First, we created a fiber configuration by means of a rule-based algorithm [1]. This geometry served as a reference for our computations. Second, we mapped the experimental fiber configuration obtained from DTI to our wedge using interpolation by inverse distance weighting. Lastly, additional to the experimental fiber configuration obtained from DTI, we included an interface surrounding the triangular pattern where the fibers are oriented in the base to apex direction. This interface was rendered non-conducting in order to mimic the presence of collagen. Nine electrodes were positioned outside the geometry to calculate extracellular potentials. For every geometry, three stimulation protocols were considered: from top to bottom, left to right, and front to back of the wedge, respectively. Electrical activity was modeled with the monodomain formulation, using the ten Tusscher ionic model [2]. The time step used was 20 μs with a temporal output of 1 ms for all simulations. The total duration of each simulation was 450 ms. The model was numerically solved using the ﬁnite element method via the Cardiac Arrhythmia Research Package (CARPentry) software [3,4], built upon extensions of the openCARP EP framework (http://www.opencarp.org) [5]. Extracellular potentials were recovered in each of the 9 electrodes from volume currents [6]. In all the simulations, local activation times (LATs) were computed.

References:

1. Bayer, J.D., et al., A novel rule-based algorithm for assigning myocardial fiber orientation to computational heart models. Ann Biomed Eng, 2012. 40(10): p. 2243-54.

2. ten Tusscher, K.H. and A.V. Panfilov, Alternans and spiral breakup in a human ventricular tissue model. Am J Physiol Heart Circ Physiol, 2006. 291(3): p. H1088-100.

3. Augustin, C.M., et al., Anatomically accurate high resolution modeling of human whole heart electromechanics: A strongly scalable algebraic multigrid solver method for nonlinear deformation. J Comput Phys, 2016. 305: p. 622-646.

4. Vigmond, E.J., et al., Solvers for the cardiac bidomain equations. Prog Biophys Mol Biol, 2008. 96(1-3): p. 3-18.

5. Plank, G., et al., The openCARP simulation environment for cardiac electrophysiology. Comput Methods Programs Biomed, 2021. 208: p. 106223.

6. Neic, A., et al., Efficient computation of electrograms and ECGs in human whole heart simulations using a reaction-eikonal model. J Comput Phys, 2017. 346: p. 191-211.

**Supplementary figures**


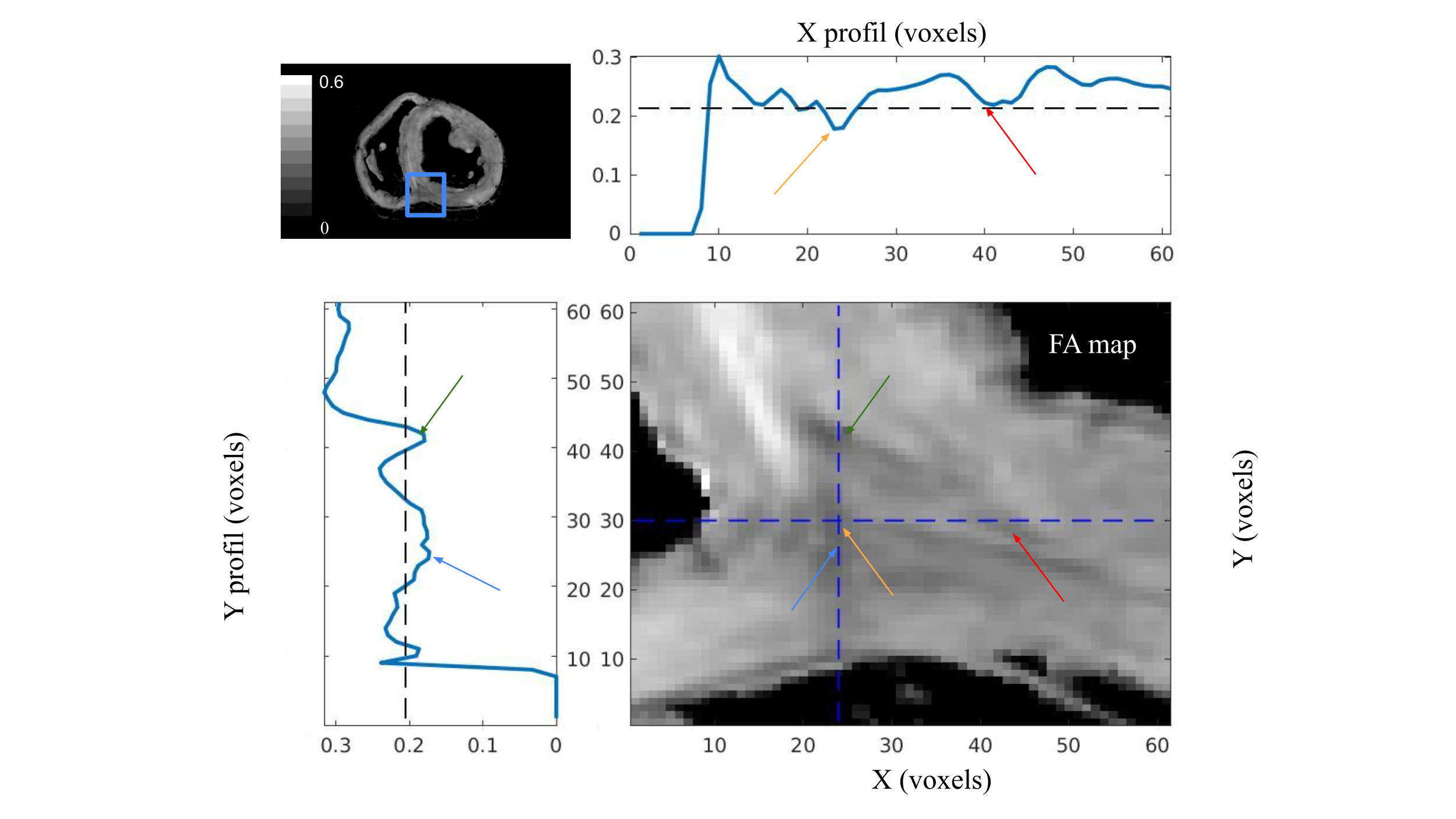


**Supp. Fig. 1:  FA profile of the basal inferoseptal left ventricular endocardium from sheep heart #5.** The FA map (between 0.1 and 0.6) is plotted with horizontal and vertical profiles (blue dotted lines) in adjacent panels. Red arrows point to the interface of the singularity with reduced FA. The black dotted line on each profile corresponds to the average of each FA profile, respectively 0.21 and 0.20 for the X profile and the Y profile.


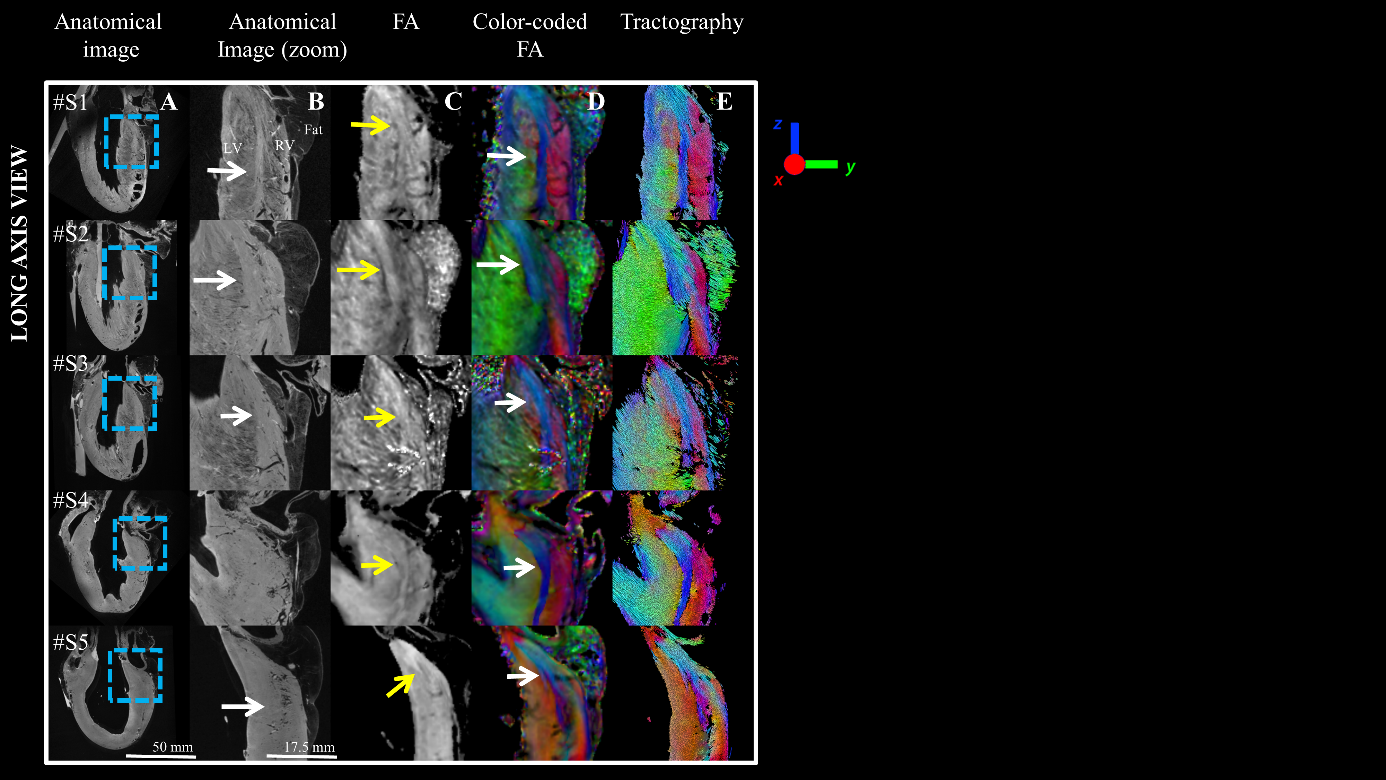


**Supp. Fig. 2: Comparison across ex-vivo sheep hearts (N=5) of the fiber orientation in the basal inferoseptal left ventricular endocardium in long axis view using anatomical and diffusion tensor images.** The legend of the metrics of Fig. 3 applies.


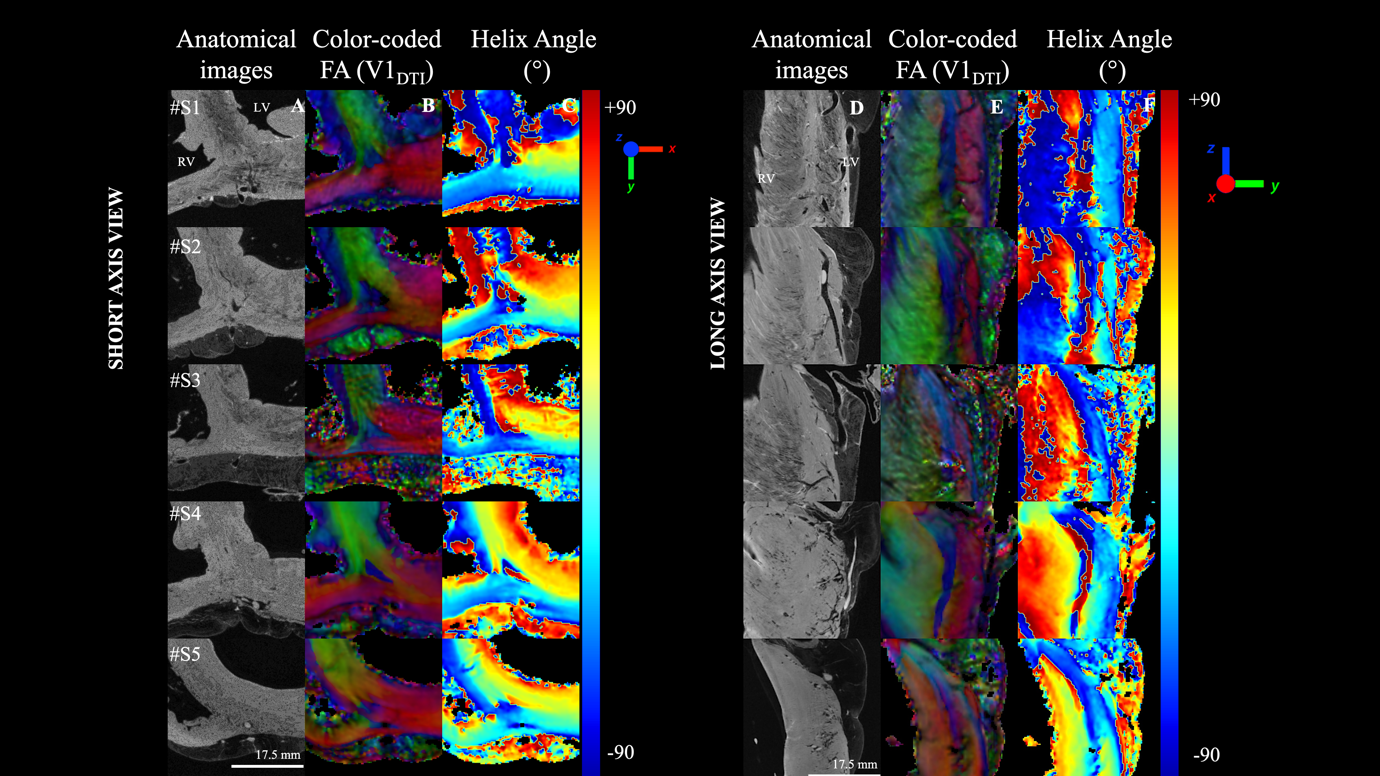


**Supp. Fig. 3: Visualization of the basal inferoseptal (BIS) left ventricular myocardium in the basal area across ex-vivo sheep hearts (N=5).** Anatomical (A, D), cFA (B, D) and Helix Angles (C, F) in short axis (left panel) and long axis (right panel) view. Using cFA maps, smooth transition of cardiomyocyte orientation is visible (gradient of green to purple) in the endocardial part of the LV while cardiomyocyte orientation changes abruptly between adjacent voxels in the IVS and in the middle of the RVIP (yellow arrow) depicting a triangular shape in the coronal view. Helix angle maps are more difficult to interpret due to wrapping but HA changes are visible in the middle of the RVIP.


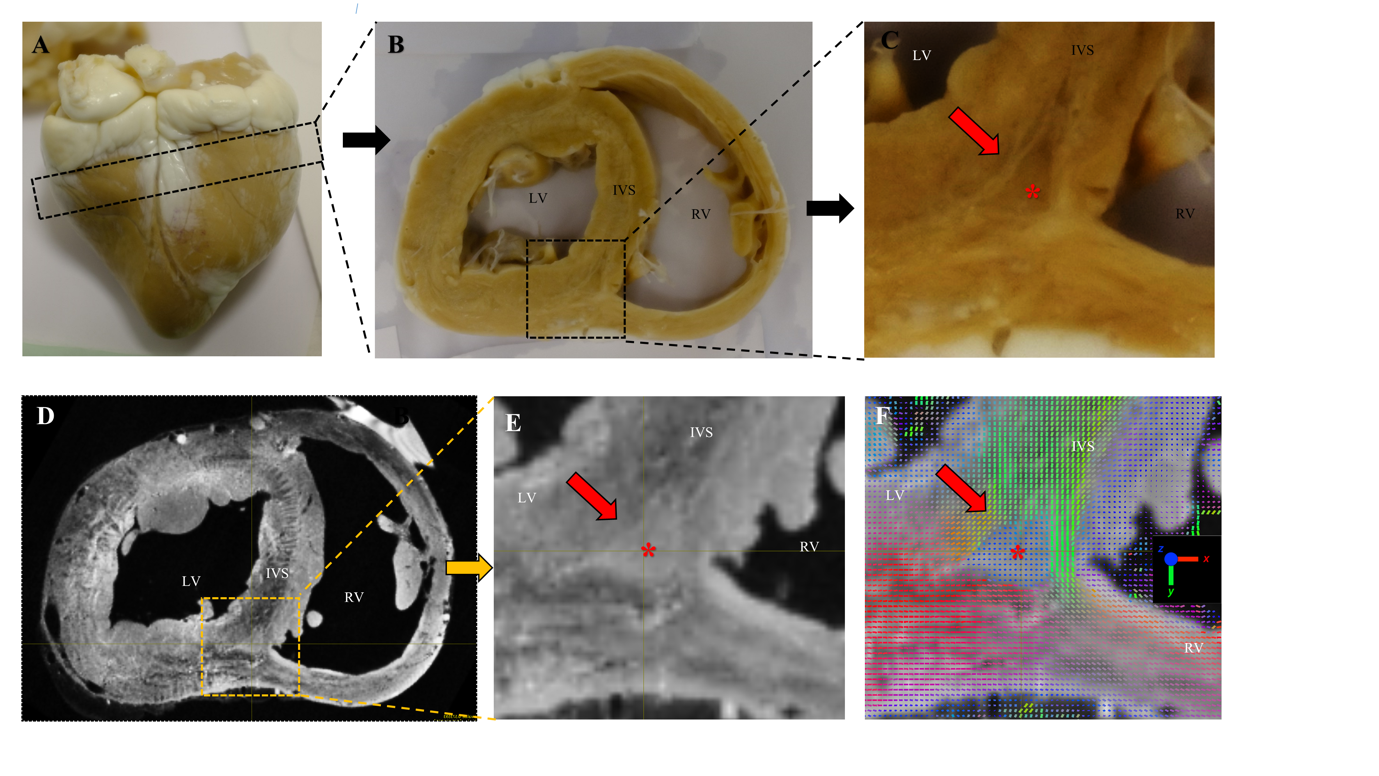


**Supp. Fig. 4:** **Macroscopic examination of the basal inferoseptal (BIS) left ventricular myocardium.** Photograph of the sheep heart #S1 before (A) and after (B) transection. C) Zoom view in the posterior wall. The red arrow and asterisk indicate the location of region depicting a triangular shape. Anatomical images (D) and zoom view (E) at roughly the same location. The cFA maps (F) encode the x-y-z coordinates of the cardiomyocyte direction derived from the DW images using a color code.


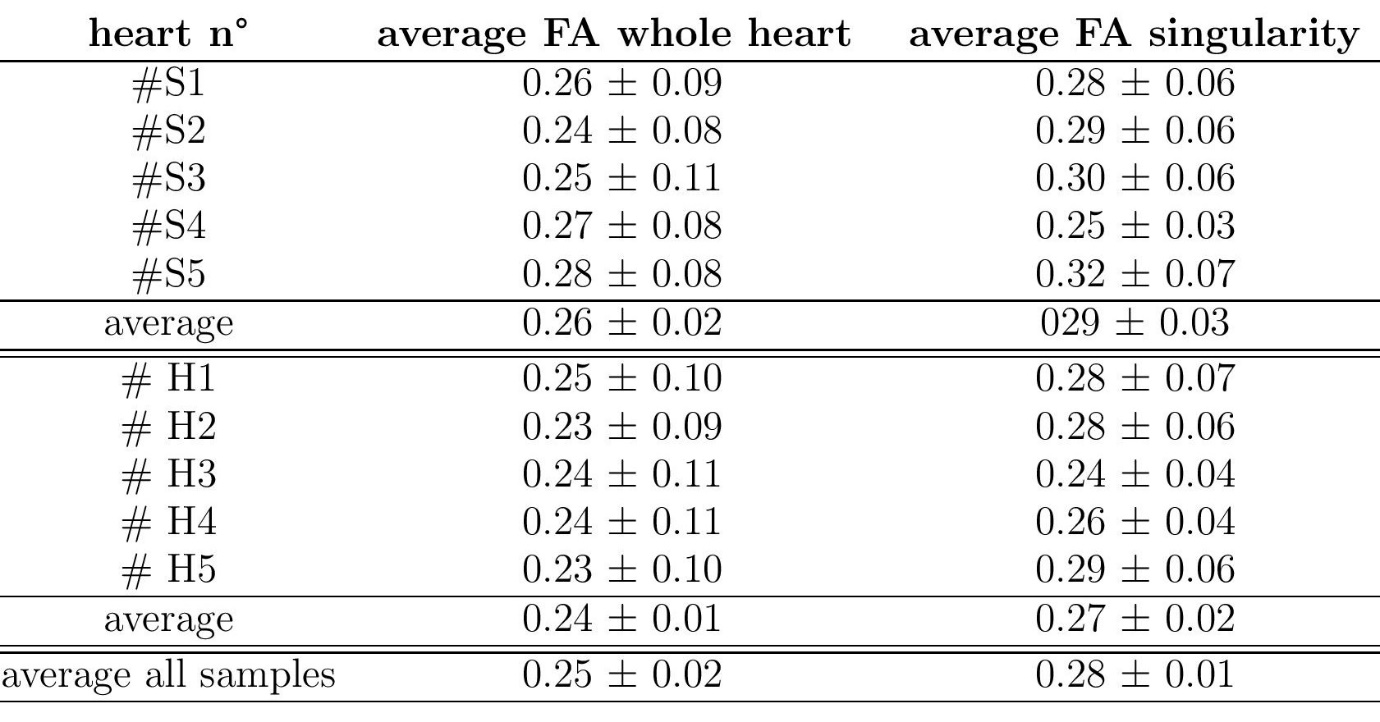


**Supplementary Table 1: Average FA in the whole heart (second column) and in the singularity (third column) on sheep (top) and human (bottom) samples.**


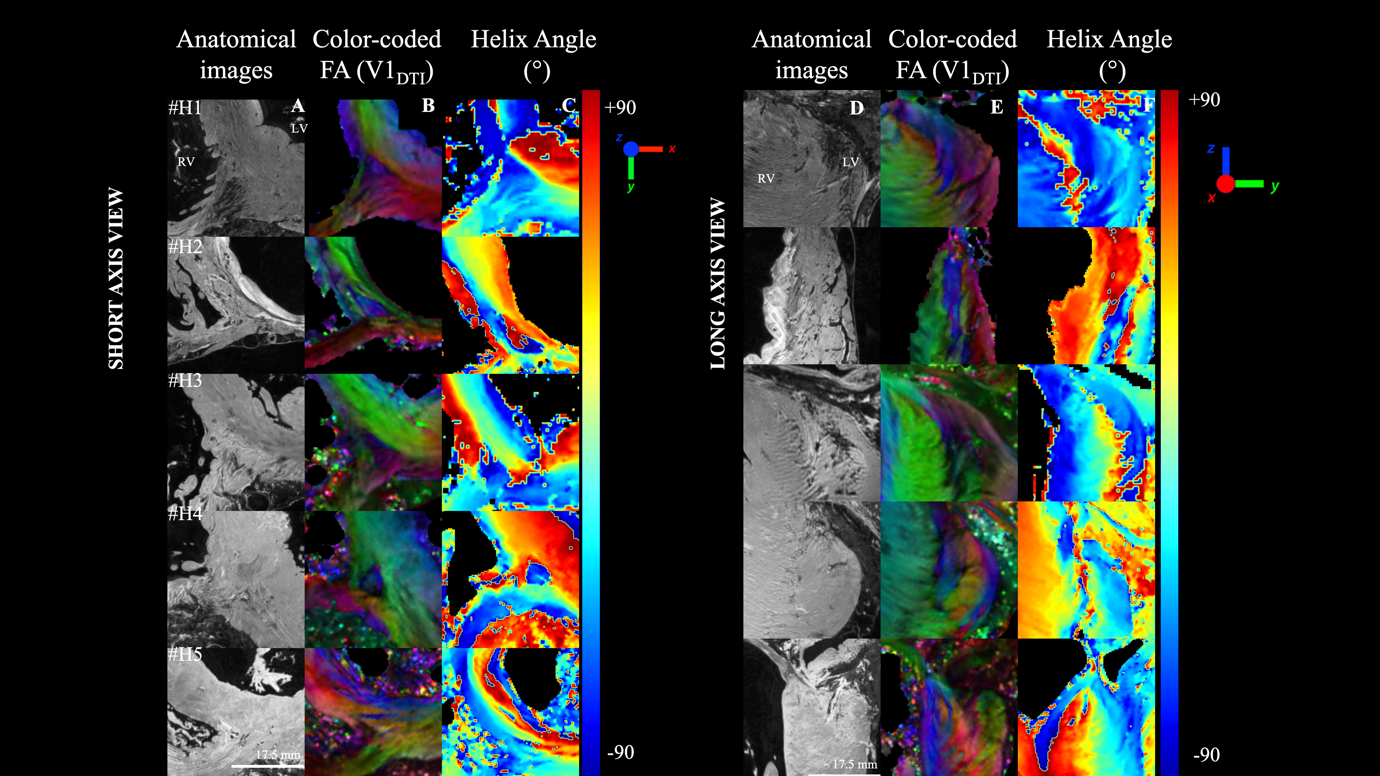


**Supp. Fig. 5: Visualization of the basal inferoseptal (BIS) left ventricular myocardium in the basal area across ex-vivo sheep hearts (N=5).** Anatomical (A, D), cFA (B, D) and Helix Angles (C, F) in short axis (left panel) and long axis (right panel) view. Using cFA maps, smooth transition of cardiomyocyte orientation is visible (gradient of green to purple) in the endocardial part of the LV while cardiomyocyte orientation changes abruptly between adjacent voxels in the IVS and in the middle of the RVIP (yellow arrow) depicting a triangular shape in the coronal view. Helix angle maps are more difficult to interpret due to wrapping but HA changes are visible in the middle of the RVIP.


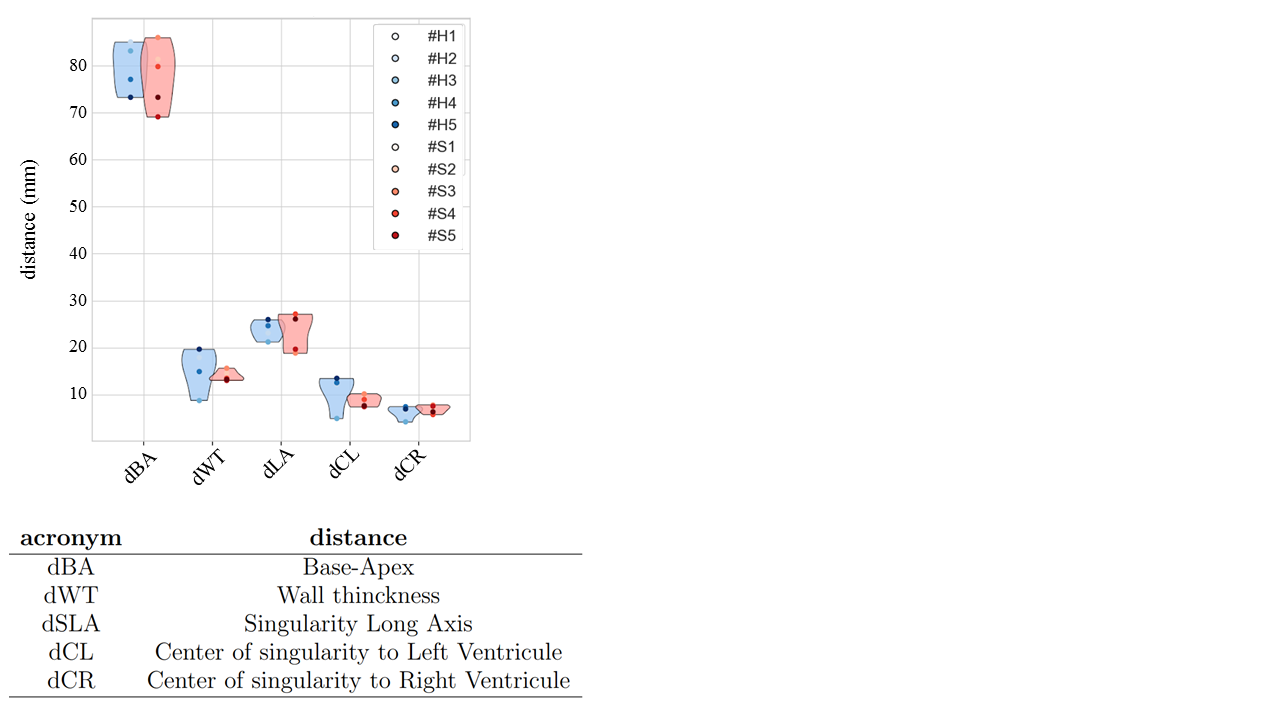


**Supp. Fig. 6: Distances measurements for ex-vivo sheep and human hearts.** (Top) Distance measurements, (bottom) legend. Each distance is described in Supplementary Figure 1.


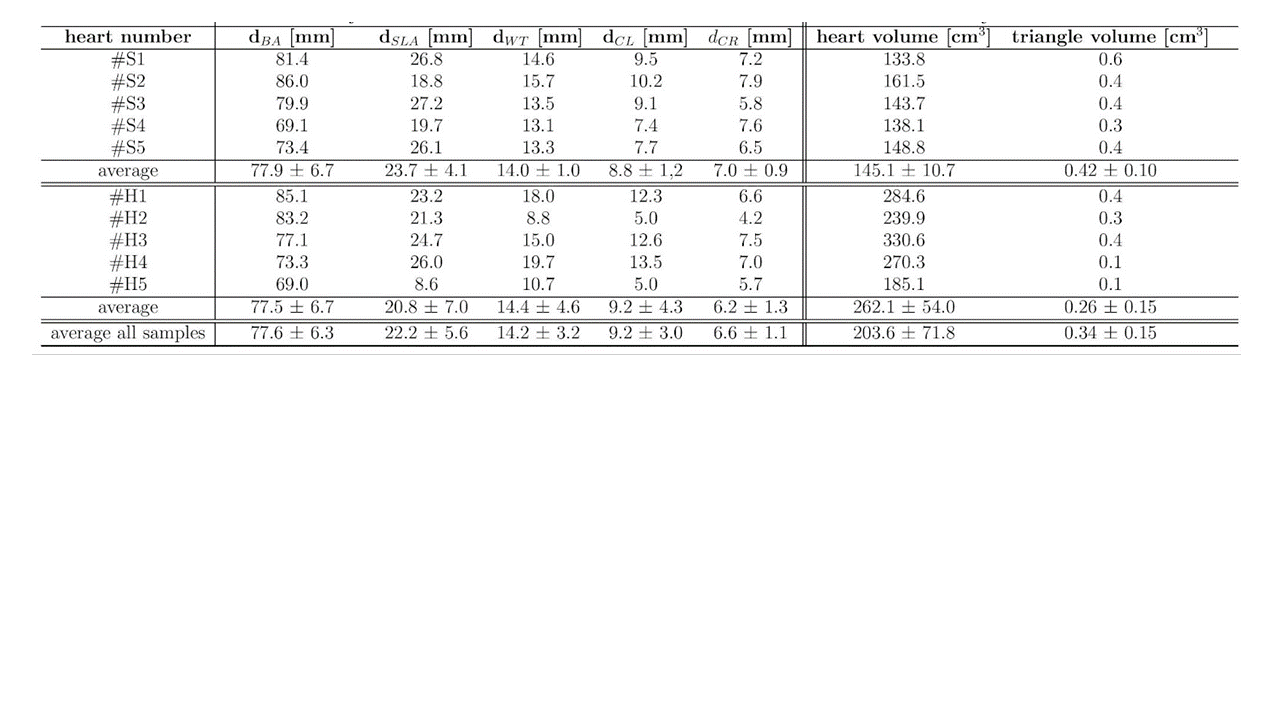


**Supplementary Table 2: Distances and volume measurements for ex-vivo sheep (top) and human (bottom) hearts.**


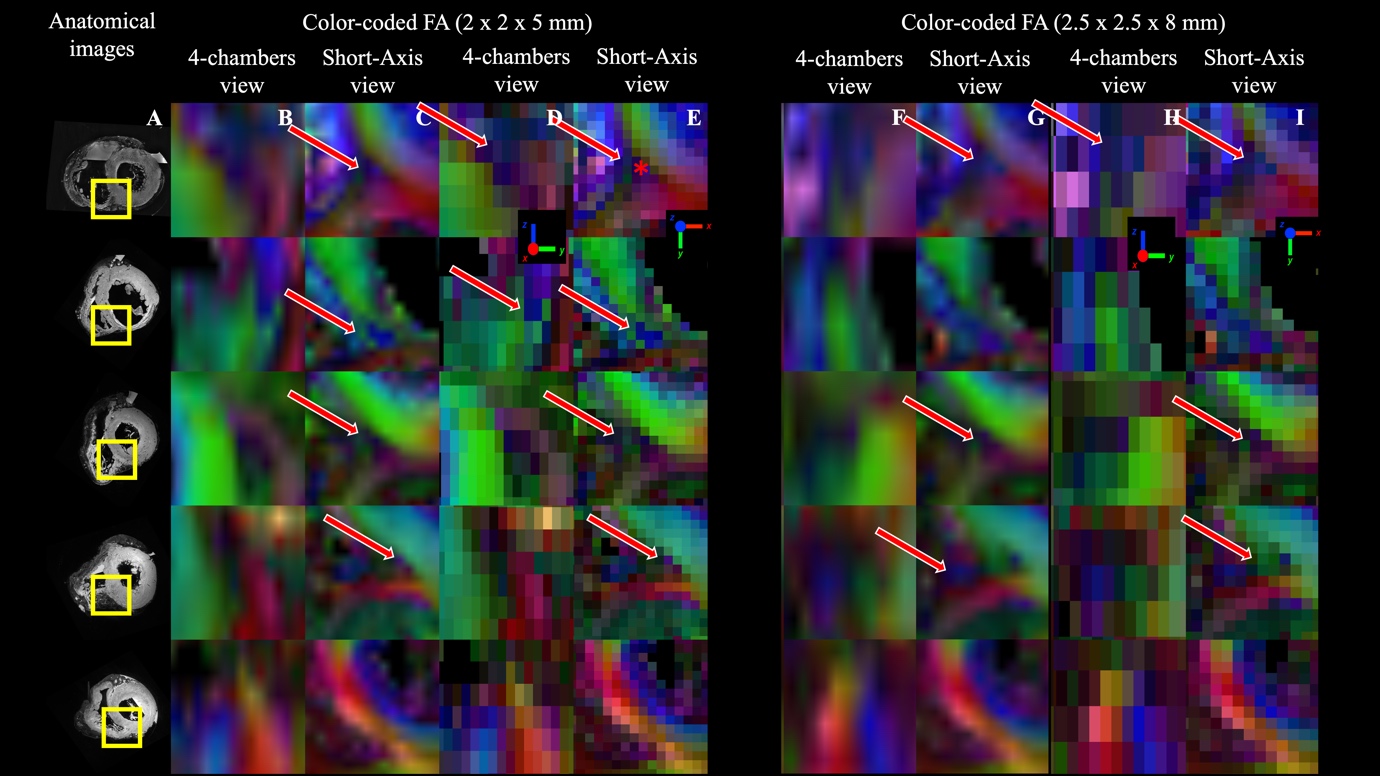


**Supp. Fig. 7: Visualization of the basal inferoseptal (BIS) left ventricular myocardium in the basal area with retrospective sampling at standard in-vivo resolution**. cFA maps in long axis and short axis view are shown for all human samples at 2.0x2.0x5.0 mm^3^ (left panel) and 2.5×2.5×8 mm^3^ (right panel) voxel resolution. Each panel show an enlarged anatomical view (A) and cFA maps in zoom-views with (B, C, F, G) and without (D, E, H, I) display interpolation. A few remaining voxels (<10 per sample, indicated by red arrows) show an abrupt cardiomyocyte orientation changes between adjacent voxels in the IVS with orientation in base to apex direction in the triangular pattern.


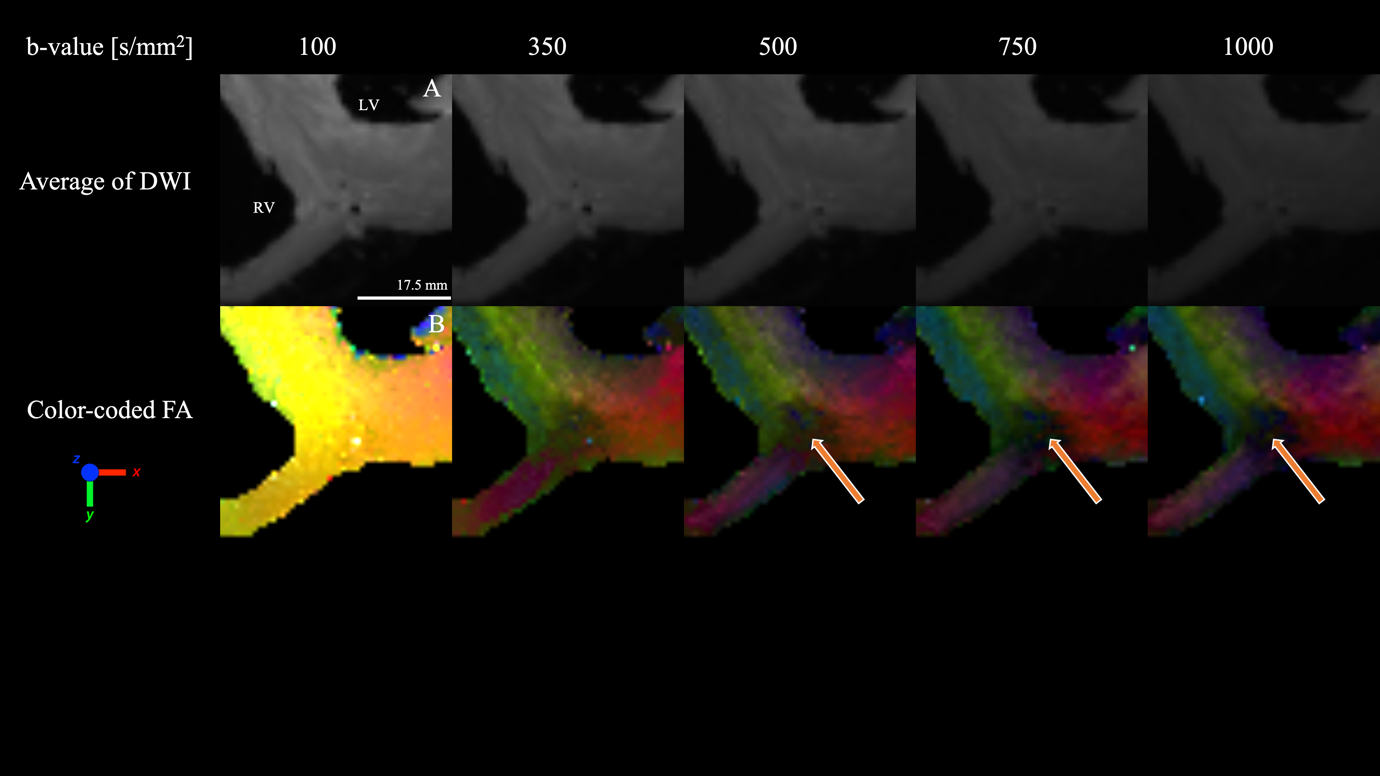


**Supp. Fig. 8: Impact of b-value on cFA maps and average DWI in the BIS area on sheep heart (#S2) in SA view.** (Top, A) Averaging of the 6 DWI directions, (Bottom, B) cFA maps. Orange arrows pointed on the aggregate cardiomyocytes with base-apex direction in the myocardium.


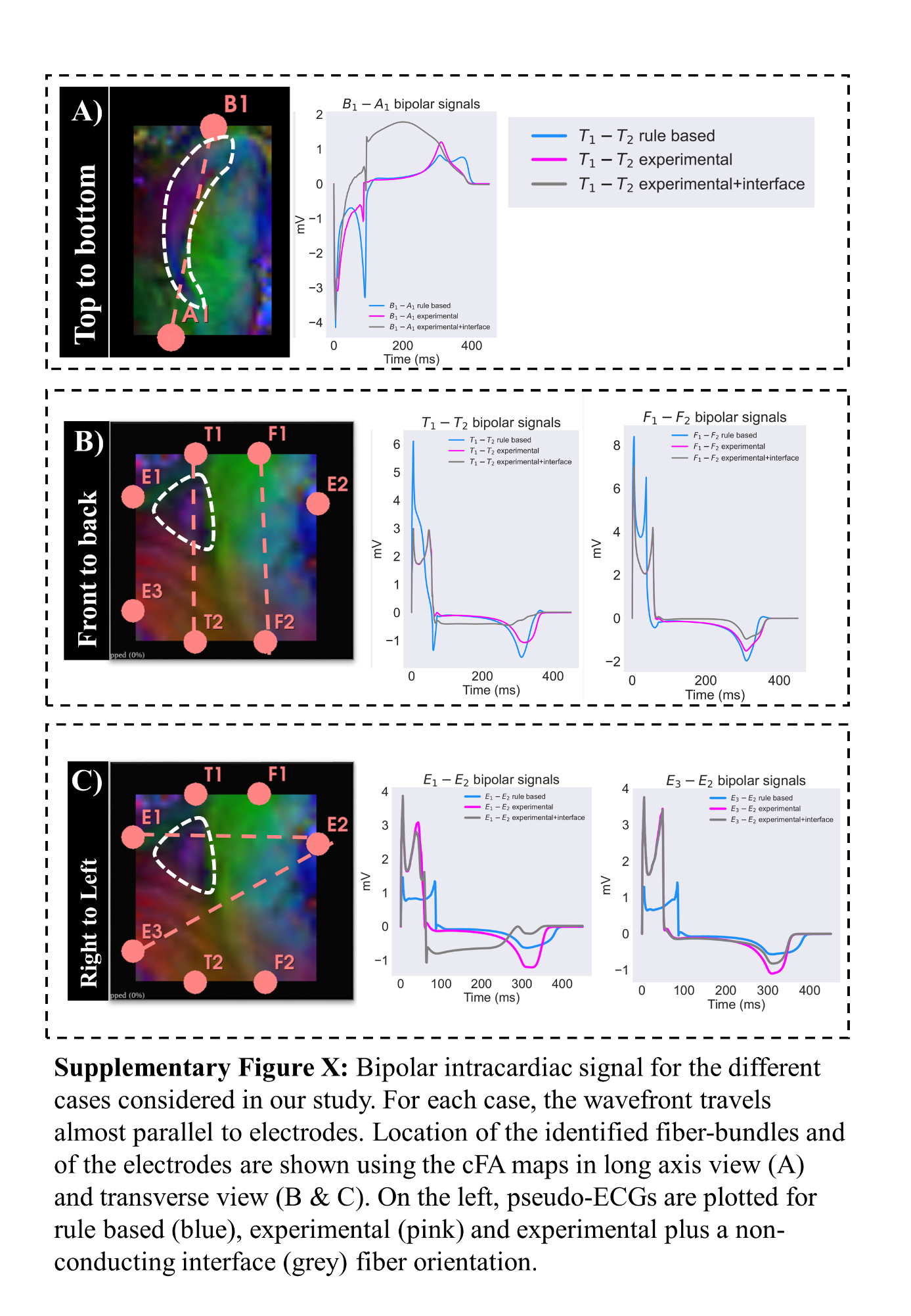


**Supp. Fig. 9: Pseudo-ECGs for the different cases considered in our study.** In each case, the wavefront travels almost perpendicular to the electrodes. The location of the identified fiber-bundles and of the electrodes are shown using the cFA maps in long axis view (A) and transverse view (B & C). On the left, pseudo-ECGs are plotted for rule-based (blue), experimental (pink) and experimental plus a non-conducting interface (gray) fiber orientation.
